# Sources, Ionic Composition and Acidic Properties of Bulk and Wet Atmospheric Deposition in the Eastern Middle Adriatic Region

**DOI:** 10.3390/toxics11070551

**Published:** 2023-06-23

**Authors:** Valentina Gluščić, Silva Žužul, Gordana Pehnec, Ivana Jakovljević, Iva Smoljo, Ranka Godec, Ivan Bešlić, Andrea Milinković, Saranda Bakija Alempijević, Sanja Frka

**Affiliations:** 1 Environmental Hygiene Unit, Institute for Medical Research and Occupational Health, Ksaverska Cesta 2, 10000 Zagreb, Croatia; vgluscic@imi.hr (V.G.); gpehnec@imi.hr (G.P.); ijakovljevic@imi.hr (I.J.); ismoljo@imi.hr (I.S.); rgodec@imi.hr (R.G.); ibeslic@imi.hr (I.B.); 2 Division for Marine and Environmental Research, Ruđer Bošković Institute, Bijenička Cesta 54, 10000 Zagreb, Croatia; amilink@irb.hr (A.M.); saranda.bakija.alempijevic@irb.hr (S.B.A.); sanja.frka.milosavljevic@irb.hr (S.F.)

**Keywords:** major ions, deposited matter, dust intrusion, open-fire events

## Abstract

Atmospheric bulk and wet deposition samples were collected simultaneously at the background coastal site in the Eastern Middle Adriatic region in order to assess the impact of major ions (Cl^−^, NO_3_^−^, SO_4_^2−^, Na^+^, K^+^, NH_4_^+^, Mg^2+^, Ca^2+^) on deposition acidity and distinguish the main sources. Higher ion levels were observed during the cold period, especially for Cl^−^, Na^+^, Mg^2+^ and K^+^. Dust intrusion caused significant increases in levels of Ca^2+^, Mg^2+^ and K^+^, while open-fire events increased the levels of K^+^. Deposition acidity showed seasonal differences as well as the influence of dust intrusion. Low ionic balance ratios indicated acidic deposition properties and the presence of organic anions. The highest neutralization ability was found for Ca^2+^, Na^+^ and NH_4_^+^. Several natural (marine, crustal) and anthropogenic sources were determined, as well as the formation of secondary aerosols. Wet deposition was characterized by higher contribution of sea salt fraction compared to bulk deposition and lower contribution of crustal fraction.

## 1. Introduction

Excessive anthropogenic activities such as combustion processes, agriculture, industry or traffic, as well as natural processes such as volcanic eruptions, events of open fire or dust storms are nowadays considered to be the main sources of different pollutants in air [1]. Once in the atmosphere, these pollutants are subjected to various chemical and physical reactions, which change their composition, shape and properties and then, finally, by the process of atmospheric cleaning are transported from the air to the ground as atmospheric deposition (AD) [2,3].

Some studies in southern and eastern European countries have shown that AD occurs mainly as dry deposition, while wet deposition is more frequent in northern and western European countries [4,5,6,7,8,9]. In arid regions, as in the Mediterranean area, dry deposition is often characterized by the predominance of crustal ions such as Ca^2+^, Mg^2+^ or crustal-biological as K^+^ [10,11] and the maximum dry deposition flux is usually observed when the wet deposition flux is at minimum value [12].

Different studies have associated the high content of Cl^−^, SO_4_^2−^ and NO_3_^−^, as well as organic acids (formic, acetic and oxalic) in AD with the appearance of acid rains [13,14,15,16]. In the seas and oceans, especially in those classified as “low nutrient low chlorophyll” ecosystems, AD could provide an important external source of macro- (N and P) and micronutrients (Fe) affecting the quality and quantity of organic matter (OM) produced by phytoplankton in the photic zone and altering the CO_2_ uptake [17,18,19,20]. Due to high concentrations of heavy metals or synergistic effects between various aerosol components, AD can also have toxic effects on marine phytoplankton [21,22]. Moreover, deposition acidity can increase the solubility of toxic heavy metals in seawater, which presents a potential risk for marine organisms and indirectly affects humans [23,24,25,26].

The largest portion of AD is composed of organic matter, black carbon and inorganic ions. The most dominant anions in the deposition are SO_4_^2−^ and NO_3_^−^. HCO_3_^−^ is an important anion for determining the ion balance, while alkaline ions (Ca^2+^, K^+^, Mg^2+^) participate in acidic–basic neutralization processes [7,8,27,28]. Different studies have indicated that ionic species make up a significant proportion of the total particulate matter mass and precipitation [6,15,29,30]. Primary inorganic ions (Na^+^, Cl^−^, Ca^2+^, Mg^2+^) mostly have a natural origin (soil or sea), while secondary inorganic ions (NH_4_^+^, NO_3_^−^, SO_4_^2−^) are predominantly of anthropogenic origin [6]. Many studies have shown that the origin, type, intensity, and spatial and temporal distribution of pollution sources influence the chemical composition of atmospheric deposition [31,32]. The investigation of the relationship between the emission sources and the chemical composition of AD is crucial to understanding its influence on the environment. Consequently, by determining the chemical composition of AD, the contributing pollution sources could be identified [7,9,28,33,34,35,36].

The Mediterranean Basin, because of its specific location between Europe, Asia and Africa, is the intersection of air masses from different regions, and consequently under the combined influence of different pollution sources. During summer, the occurrence and recurrence of forest fires as well as high intensity of photochemical processes makes deposition phenomenology more complex. Moreover, during summer in the western and central Mediterranean area, intrusion of Sahara dust particles is frequent, while in the eastern area dust outbreaks are frequent during autumn and spring [5].

In the eastern middle Adriatic coastal region, studies that investigate inorganic content of AD are scarce, and even the deposition fluxes of major ions in bulk and wet AD are largely unexplored. Čačković et al. [37] monitored AD at three locations in Šibenik-Knin County, Croatia, from 1999 to 2004. The total (bulk) deposition matter was determined, as well as the content of some acidic anions (F^−^, Cl^−^, NO_3_^−^, SO_4_^2−^) and heavy metals (Pb, Cd, Tl). Jakovljević et al. [38] reported the first data of organic pollutants (PAHs and nitroaromatic compounds) in aerosols and in AD and their temporal variabilities over the central Adriatic coast area. Orlović-Leko et al. [39] investigated dissolved organic carbon, surface active substances and copper complexing capacity in bulk precipitation in the Šibenik area, periodically, from 2003 to 2007. Other studies in the area (Šibenik, Split, Rogoznica) included measurements of airborne particulate matter, PM_2.5_ and PM_10_, [40], inorganic and organic ions in PM_2.5_ [41], surface active substances in PM_2.5_ [42] and PAHs in PM_10_ [43]. All these studies emphasized the complexity of air pollution in the middle Adriatic region and highlighted the presence of the combined influence of local, regional and long-range natural and anthropogenic pollution sources as well as the need for further studies addressing air quality and pollution source apportionment in this area in an effort to mitigate and adapt to climate change.

To our best knowledge, there have been no studies in the area that were specifically focused on the concentrations of major ions in AD, or their balance, interactions and sources. Thus, the AD acidic properties and its possible influence on the environment due to acidification, neutralization or nutrient enrichment, which are extremely important in an oligotrophic environment such as the middle Adriatic Sea area, are relatively unknown.

Therefore, an extensive field campaign over a six-month period was conducted within the BiREADI project in the eastern middle Adriatic region to make progress in the fundamental understanding of the linkage between AD inputs and complex sea surface biochemical responses by observation and modelling. During the same campaign, N and P wet deposition fluxes [20] as well as concentrations of trace metals in bulk deposition samples [26] were studied to assess the extent to which deposition fluxes of external nutrients and potentially toxic metals can affect the biology and chemistry of sea surface layers at the Adriatic coastal area. This research is focused on a data set comprising major ions in bulk and wet deposition collected as part of BiREADI’s field sampling, to gain better insight into the seasonal variabilities of AD ionic composition, dominant sources and acidic properties. The data were analyzed with respect to certain special events that typically occur in coastal Mediterranean environments, such as desert dust intrusion and local/regional open-fire events. The fractional acidity, ionic balance, neutralization and acidification potential and neutralization factors of the measured ions were examined in order to understand the chemical nature of AD and its possible influence on the environment. In order to estimate possible pollution sources, regression analysis, triangular diagrams and enrichment factors were used.

## 2. Materials and Methods

### 2.1. Measuring Site

The measuring site Martinska (43°73′ N, 15°87′ E) is located on the eastern middle Adriatic coast, across from the city of Šibenik (Supplementary Information (SI) Figure S1). It is classified as a coastal-rural measuring site. Šibenik, the capital of the Šibenik-Knin County, is a historic city located in central Dalmatia in the wider part of the Krka river estuary with 46,332 inhabitants. It has developed tourism and mariculture especially in the summertime. In the past, metalworking activities were strong in Šibenik (iron alloys, aluminum production), but reduced after the war in the 1990s. Moreover, in the past 15 years the territory of the Šibenik-Knin County was an area on the Adriatic coast most affected by local wildfire events, especially during summer [44]. The eastern middle Adriatic region has a Mediterranean climate with mild, wet winters and hot, dry summers with a high solar radiation index.

### 2.2. Atmospheric Deposition Sampling

Bulk deposition sampling started on the 23 January and lasted until 10 July 2019, while wet deposition sampling started two weeks later, on 7 February 2019. Over the 6-month sampling period, a total of 12 bulk and 9 wet biweekly deposition samples were collected. Bulk deposition was collected using two Bergerhoff deposit gauges (without the possibility of sample cooling during the sampling time), placed next to each other, consisting of a metal stand, a bottle holder with a bird guard and an HDPE collecting bottle (diameter of 100 mm). Samplers were placed at the measuring site rooftop (~5 m above ground).

Wet deposition was collected with an automatic wet-only deposition sampler (NSA 181, Eigenbrodt, GmbH&Co, Königsmoor, Germany) placed ~2 m above the ground. When rainfall occurs, the precipitation sensor opens the sampler lid and the precipitation flows directly through a funnel (collection aperture 500 cm^2^) into an HDPE collecting bottle kept below a funnel in a dark refrigerator at a constant temperature of 4–8 °C. In order to prevent evaporation losses during sampling, the opening of the collecting bottle fitted well to the sampler lid. On days without rain, the precipitation sensor re-seals the sampler lid, which prevents dry deposition from entering the sampler.

### 2.3. Analysis of Major Ions in Atmospheric Deposition

Before analysis, wet deposition samples and samples from one Bergerhoff sampler were filtered to remove the insoluble portion of samples. The filtrate volume was measured and aliquots were used for the analysis of Cl^−^, NO_3_^−^, SO_4_^2−^ and NH_4_^+^. Anions were analyzed by ion chromatography (IC DX-120, Dionex Corporation, Sunnyvale, CA, USA) with suppressed conductivity detection. An AS14 analytical column (4 × 250 mm) with an AG14 guard column (4 × 50 mm) under isocratic elution (3.5 mM Na_2_CO_3_/1 mM NaHCO_3_) was used for separation. By diluting the multi-element standard (Merck, γ(NO_3_^−^, SO_4_^2−^) = 500 mgL^−1^; γ(Cl^−^) = 250 mgL^−1^), nine calibration standard solutions were prepared and each standard was analysed in triplicate (R^2^ = 0.999). The detection limit for Cl^−^ was 0.02 mg m^−2^ d^−1^, and 0.04 mg m^−2^ d^−1^ for NO_3_^−^ and SO_4_^2−^ [37].

NH_4_^+^ was determined spectrophotometrically on a Super Aquarius 9200 Spectrophotometer (Cecil Instrumentation, Cambridge, UK) by the Nessler method [45]. Calibration standard solutions were prepared by diluting the certified standard solution (Sigma-Aldrich, γ (NH_4_^+^) = 1000 mg L^−1^). Milli-Q water was used as a blank and was treated in the same way as wet deposition samples. The detection limit for NH_4_^+^ was 0.12 mg m^−2^ d^−1^.

Content of Na, Mg, K and Ca in bulk and wet deposition samples was determined by inductively coupled plasma mass spectrometry (ICP-MS 7500cx, Agilent Technologies, Waldbroon, Germany). Bulk deposition samples were quantitatively transferred to vessels and evaporated to dryness and the bulk deposition amount was determined by gravimetry. Samples were further digested with nitric acid (65% p.a. Merck) and microwaves (1000 W) in an UltraCLAVE digestion system (Milestone Srl, Milan, Italy). Wet deposition samples were acidified with nitric acid to a final content of 5% (*v*/*v*) of nitric acid. All elements were analyzed in collision mode with helium gas and Sc, Ge and Rh were used as internal standard solutions. Accuracy was checked in each batch with certified reference materials (NIST 1643e, ERM CZ 120 and NIST 1648a).

### 2.4. pH Measurements

The pH was measured using a pH meter (Type 691, Metrohm AG, Herisau, Switzerland), equipped with a combined glass electrode for measurements of samples of low ionic strength. Due to omission, pH was measured in samples starting from 1 April 2019.

### 2.5. Meteorological Parameters and Data Analysis

**Figure 1:**
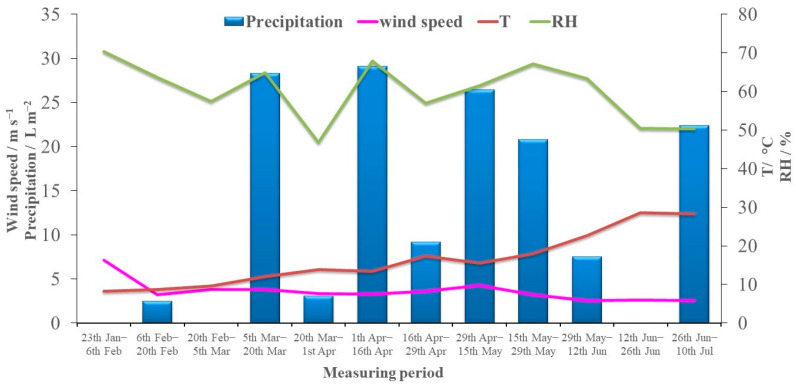
Average temperature, humidity, precipitation and wind speed determined during the sampling period in 2019.

Official real-time meteorological data were obtained from the Croatian Meteorological and Hydrological Service (CMHS) at the nearby station in Šibenik. Temperature, relative humidity, amount of precipitation and wind speed were recorded every minute of each sampling day and the average biweekly values are shown in Figure 1. The average temperature ranged from 8.5 °C to 28.5 °C, while relative humidity ranged from 46.8% to 70.0% and precipitation from 0 L m^−2^ to 29.1 L m^−2^. Based on the official real-time measured temperature values from the CMHS and in accordance with the South East European Virtual Climate Change Center (SEEVCCC) web site (http://www.seevccc.rs, accessed on 5 April 2023), in further data processing the obtained levels of pollutants in bulk and wet deposition were divided into cold (February–April) and warm period (May–July) (SI Figure S2). The average temperature in the cold period was 10.9 °C (range was from 8.3 °C to 13.8 °C) and in the warm 21.7 °C (range from 15.6 °C to 28.5 °C). Average precipitation during the cold period was 12.6 L m^−2^ and during the warm period it was slightly higher, 14.4 L m^−2^. Average relative humidity during the cold period was 61.9% and during the warm period 58.3%.

According to on-line data about local fire events in the vicinity (~20 km) of sampling site Martinska, provided by the Fire department of the Šibenik-Knin County (https://www.vatrogastvo-sibenik-knin.hr/, accessed on 5 April 2023), during the 6-month sampling period, fire events of characteristic low Mediterranean vegetation with holm oak, pine and cypress forests, as well as fires at local wild waste disposal sites were recorded in cold as well as in the warm period. In the cold period, several open-fire events were recorded from the 16 until 21 February and from 31 March until 2 April, while in the warm period only one event was recorded from 6 until 15 June 2019 as reported in our previous publication [20,26,38]. According to the data given by the Croatian Meteorological and Hydrological Service [46] and South East European Virtual Climate Change Center (SEEVCCC), the strong southwest air masses from North Africa brought Saharan dust particles into the higher layers of the atmosphere in the period from 21–25 April (SI Figure S3). In further data analysis, samples collected during open-fire events or desert dust intrusion events were excluded from the statistics for cold and warm periods, and were presented separately.

The STATISTICA 14 software package (Tibco Software Inc., Palo Alto, CA, USA) was used for statistical analysis. Regression analysis included determination of Pearson’s correlation coefficients and multiple linear regression analysis (MLR). Regression coefficients (b) and standardised regression coefficients (β) were calculated, together with corresponding standard errors, levels of significance and coefficients of determination. Triangular diagrams were created using Grapher Golden software.

## 3. Results and Discussion

### 3.1. Major Ions in Bulk Deposition

**Figure 2:**
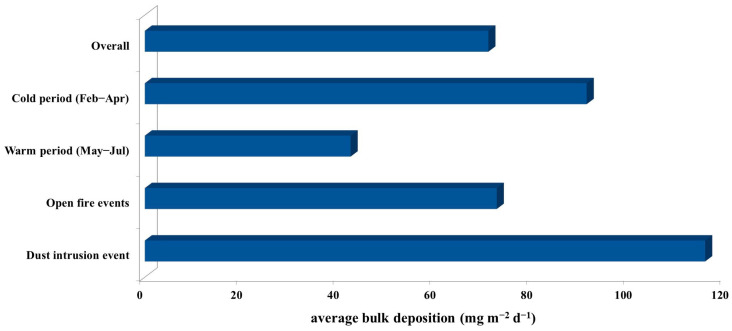
Average bulk deposition fluxes during the sampling campaign from February to July 2019 at the Middle Adriatic coastal site.

Descriptive statistics parameters for bulk deposition levels and ionic content of bulk deposition, are presented in Table 1. For better comparison with literature data, results are expressed as deposition fluxes in mass concentrations (mg m^−2^ d^−1^), as well as equivalent concentrations in μeq L^−1^. Figure 2 shows average bulk deposition fluxes in cold and warm period and during specific events (open fires and desert dust intrusion).

The observed bulk deposition fluxes were low and ranged from 22 mg m^−2^ d^−1^ to 185 mg m^−2^ d^−1^ with an average of 71 mg m^−2^ d^−1^. The annual limit value of 350 mg m^−2^ d^−1^ given by the Croatian Regulation on Levels of Pollutants in Ambient Air [47] was not exceeded during the studied period. A similar level (73 mg m^−2^ d^−1^) was also found in previous research at the eastern middle Adriatic, on the island of Žakan in the Kornati archipelago [37]. Our results are also comparable with the results obtained in background and rural coastal sites such as Isle of Mallorca in the western Mediterranean, with a deposition flux of 62 mg m^−2^ d^−1^ [9] and 46 mg m^−2^ d^−1^ in Cienfuegos Province at a Caribbean coast [1]. Clear seasonal differences in bulk deposition fluxes were found between cold and warm periods (Figure 2), with higher values during the cold period. Results for the cold period (91 mg m^−2^ d^−1^) are comparable with findings reported for autumn and winter season (93.1 mg m^−2^ d^−1^ and 88.6 mg m^−2^ d^−1^, respectively) at a measuring station located in the Balearic Islands (Spain, Western Mediterranean) by Cerro et al. [9]. Results obtained for the warm period (43 mg m^−2^ d^−1^) were higher than the results reported for spring (30.2 mg m^−2^ d^−1^) and comparable with results reported for summer (40.5 mg m^−2^ d^−1^) in the previously mentioned study by Cerro et al. [9] at the regional background measuring station.

The average deposition fluxes obtained for samples collected during and/or after fire events are similar to the average value for the overall period (Figure 2). The highest bulk flux (185 mg m^−2^ d^−1^) was determined at the beginning of April when the intense fire event occurred followed by high precipitation. Due to low rainfall between the 6 and 20 February and between the 6 and 15 June, when other fire events occurred, the bulk deposition fluxes were not as prominent as in samples from April. It is important to note that according to official CMHS measurements at the nearby Šibenik station, in April the precipitation amount was above the multi-year average. Moreover, the observed fire events in winter and summer have spread on two sampling periods, e.g., one fire episode started a few days before the end of the sampling of the first sample and ended a few days after the beginning of the second sample period. In general, deposition flux levels affected by emissions from fire episodes in different seasons could have been influenced by the combined effects of several factors such as fire intensity, distance from the sampling site, the attacked surface area, type of burning material, as well as the local availability of precipitation. The obtained average bulk deposition flux during the dust intrusion was 116 mg m^−2^ d^−1^ which is almost twice the average for the overall period and significantly higher than in the warm period. A study in the Mallorca Isle found that Sahara dust intrusion events can contribute to much higher deposition rates of 501 mg m^−2^ d^−1^ [9]. The influence depends on the source distance or the origin of the air masses. Furthermore, some studies showed that sampling sites in the western Mediterranean have twice as high bulk deposition fluxes in comparison to sampling sites in the eastern Mediterranean due to strong and frequent Saharan dust episodes [23]. The occurrence of Sahara dust episodes over central Italy are especially frequent during the summer [5]. In the southeastern Adriatic region, Đorđević et al. [48] found that the highest amount and frequencies of precipitation were related to air masses coming from Africa and the western and central Mediterranean area. The results of this study highlighted that short and intense specific events have significant influence on the amount of atmospheric deposition as well as seasonal distribution since the occurrence of these intensive events has a seasonal pattern.

The determined daily fluxes of total inorganic anions in bulk deposition at Martinska site ranged from 0.20 mg m^−2^ d^−1^ to 19.44 mg m^−2^ d^−1^, while total cations ranged from 0.11 mg m^−2^ d^−1^ to 10.19 mg m^−2^ d^−1^ (Table 1). For the overall measuring period, average deposition fluxes (in mg m^−2^ d^−1^) followed the order Cl^−^ > NO_3_^−^ > Na^+^ > Ca^2+^ > SO_4_^2−^ > NH_4_^+^ > K^+^ > Mg^2+^. Equivalent concentrations of measured ions in the same samples ranged from 1.31 μeq L^−1^ to 265.07 μeq L^−1^, with average values decreasing in the order Ca^2+^ > Cl^−^ > Na^+^ > NH_4_^+^ > NO_3_^−^ > SO_4_^2−^ > Mg^2+^ > K^+^ (Table 1). Among the anions, Cl^−^ was the predominant ion, while the most abundant cations were Ca^2+^, Na^+^ and NH_4_^+^. The determined ion fluxes (mg m^−2^ d^−1^) were lower than values reported for industrial and residential sites but similar to those reported at a background station in Aliaga region in Turkey [49] as well as in a rural area of Cuba [1]. Rossini et al. [50] found much higher fluxes of ions at industrial, urban and a remote areas in the Venice Lagoon (northwest Adriatic). Izquierdo and Avila [11] previously reported a lower concentration range for ions in bulk deposition content at a rural background site in northeastern Spain in a period of 2.5 years, while Pieri et al. [34] found a higher concentration range for ions determined in bulk deposition content at one urban and one rural site in the southern Po Valley (northern Italy) over a 3-year sampling period. The most abundant cation in that survey at both sampling sites was Ca^2+^, followed by Na^+^ and NH_4_^+^, while the most abundant anion was SO_4_^2−^. Determined ion deposition fluxes (mg m^−2^ d^−1^) and ion equivalent concentrations (µeq L^−1^) in bulk and wet deposition content in comparison with literature data are presented in SI Table S1.

**Figure 3:**
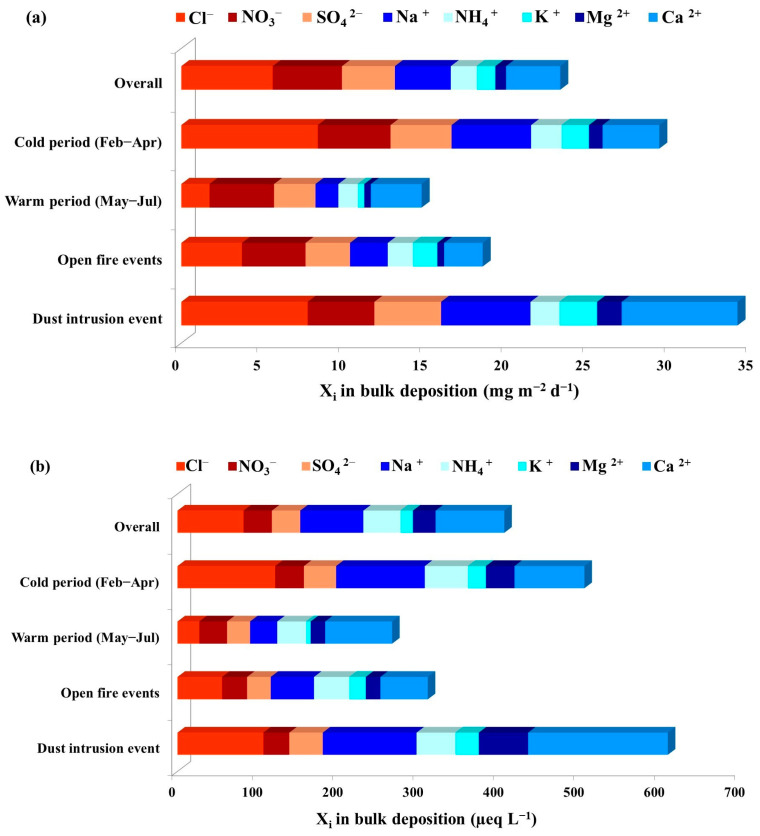
Average (**a**) deposition fluxes and (**b**) equivalent concentrations of ions in bulk deposition during the sampling campaign from February to July 2019 at the Middle Adriatic coastal site.

A clear seasonal difference was found between deposition fluxes and equivalent concentrations of ions determined in the cold and warm sampling period (Figure 3). For all ions except Ca^2+^, higher average fluxes (in mg m^−2^ d^−1^) were determined during the cold period; however, the difference was statistically significant only for Cl^−^, Na^+^ and K^+^. Average deposition fluxes (in mg m^−2^ d^−1^) during the cold period were in the order Cl^−^ > Na^+^ > NO_3_^−^ > SO_4_^2−^ > Ca^2+^ > NH_4_^+^ > K^+^ > Mg^2+^, while for the warm period the order was NO_3_^−^ > Ca^2+^ > SO_4_^2−^ > Cl^−^ > Na^+^ > NH_4_^+^ > Mg^2+^ > K^+^. The order for average equivalent concentrations (in µeq L^−1^) slightly differed, with lower values of nitrates and sulphates; being Cl^−^ > Na^+^ > Ca^2+^ > NH_4_^+^ > SO_4_^2−^ > NO_3_^−^ > Mg^2+^ > K^+^ during the cold period and Ca^2+^ > NH_4_^+^ > NO_3_^−^ > Na^+^ > SO_4_^2−^ > Cl^−^ > Mg^2+^ > K^+^ during the warm period. It is evident that levels of Na^+^ and Cl^−^, typical for the marine environment, were higher during the cold period. Indeed, in marine environments, more turbulent weather conditions during winter and spring periods are expected to enhance the primary emission of sea-salt aerosols because of the friction effect of the wind on the sea surface [51] in contrast to summer, which is characterized by weaker winds and a calmer sea surface.

During dust intrusion, average ion concentrations in bulk deposition ranged from 28.8 µeq L^−1^ to 173.8 µeq L^−1^ and followed the order Ca^2+^ > Na^+^ > Cl^−^ > Mg^2+^ > NH_4_^+^ > SO_4_^2−^ > NO_3_^−^ > K^+^. Deposition fluxes, as well as equivalent concentrations of all ions except NO_3_^−^, were higher than average values for the overall sampling period (Figure 3), especially for Ca^2+^, Mg^2+^ and K^+^, for which average values were more than 2 times higher. These results were not surprising, as Ca^2+^ is often used as a tracer ion for soil or mineral dust [52]. Furthermore, Cerro et al. [9] reported higher levels of Ca^2+^ in the Mallorca Isle during several dust outbreak episodes from the Sahara in spring, summer and winter. Samples collected during fire events were characterized by lower levels of Ca^2+^, Na^+^, Mg^2+^ and Cl^−^, and more than 30% higher values of K^+^ in comparison to the overall sampling period. These results are not surprising, since potassium is often used as an inorganic tracer for biomass burning [53]. Cvitešić Kušan et al. [41] reported that local open-fire events affected the levels of K^+^ in the PM_2.5_ particle fraction at the middle-Adriatic region.

**Figure 4:**
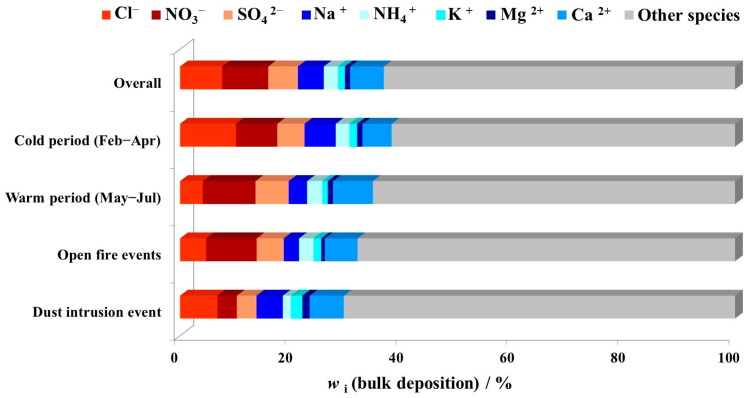
Average contributions of each ion to the bulk deposition mass during the sampling campaign from February to July 2019 at the middle Adriatic coastal site.

Figure 4 presents the average contributions of each ion to the bulk deposition mass for the overall period (calculated by mass concentrations), as well as during the cold and warm periods, and special events. During the entire sampling period, the obtained total anion flux varied from 4.6 mg m^−2^ d^−1^ to 29.2 mg m^−2^ d^−1^ with an average value of 13.1 mg m^−2^ d^−1^, which accounted for 21.2% of average bulk deposition mass. Total cation flux varied from 3.9 mg m^−2^ d^−1^ to 24.1 mg m^−2^ d^−1^ with an average of 10.2 mg m^−2^ d^−1^, which accounted for 15.5% of average bulk deposition mass (Figure 4). These values are lower than those previously reported by Tsai [27], who determined a higher contribution of anions (47.06%) and cations (52.34%) to the total bulk deposition mass at a Taiwanese coastal suburban area. The overall average contribution of each ion to the bulk deposition mass followed the order NO_3_^−^ > Cl^−^ > Ca^2+^ > SO_4_^2−^ > Na^+^ > NH_4_^+^ > K^+^ > Mg^2+^, while during seasons and special events the order of contributions changed. The cold period was characterized by an increased contribution of Cl^−^. During open-fire events, the average contributions of all ions were not significantly different from the overall values, except for NO_3_^−^ and K^+^, which slightly increased. The lowest contributions of SO_4_^2−^ and NO_3_^−^ were observed during dust intrusion. In general, the influence of open-fire events on contributions of each ion to the bulk deposition mass was not as clear as for dust intrusion, which was discussed in the chapter before.

The contribution of each anion to the average total anion flux in bulk deposition (calculated by mass concentrations) for the overall period followed the order NO_3_^−^ > Cl^−^ > SO_4_^2−^ (SI Figure S4a), while the order of the contribution of each cation to the average cation flux was Ca^2+^ > Na^+^ > NH_4_^+^ > K^+^ > Mg^2+^ (SI Figure S4b). Among the anions, the highest contribution to the total anion flux came from NO_3_^−^ with 41.5%, while Ca^2+^ ion was the most abundant cation in the total cation flux with 39.2%. Depending on the season or special events, the contribution of each anion to the total anion flux and each cation to the total cation flux followed a different order.

Among the cations, Ca^2+^, Na^+^ and NH_4_^+^ were the most predominant ions with average concentrations of 85.5 μeq L^−1^, 78.4 μeq L^−1^ and 43.6 μeq L^−1^, respectively, which accounted for 39.2%, 27.5% and 17.8% of the total determined cations in bulk deposition content (calculated by mass concentrations). The determined average concentrations (in μeq L^−1^) for K^+^ and Mg^2+^ accounted for less than 10% of the contribution of each ion to the total cation mass in bulk deposition content. Higher K^+^ and Mg^2+^ contribution to the total cation mass in bulk deposition (19.26% for sampling period of 2.5 years) was previously reported by Tsai et al. [27] at a coastal suburban area in Taiwan.

NO_3_^−^, SO_4_^2−^ and NH_4_^+^ are considered as the dominant ions of secondary inorganic aerosols and major constituents of fine particulate matter mass (PM_2.5_), which are produced in the atmosphere from their gaseous precursors (NO_x_, SO_2_, and NH_3_) in gas to particle photochemical reactions. SO_4_^2−^ ions are primarily formed through homogeneous and heterogeneous oxidations of sulfur dioxide (SO_2_) emitted from anthropogenic and natural sources. Gas-phase oxidations of sulfur dioxide (SO_2_) by the hydroxyl radical (°OH) and stabilized Criegee intermediates (sCIs), aqueous-phase reactions (in-cloud oxidation of SO_2_ by H_2_O_2_, O_3_, and O_2_), as well as heterogeneous reactions associated with aerosol water are considered to be major formation pathways for ambient SO_4_^2−^ [54]. Although the gas-phase oxidation pathway is considered slower in comparison with aqueous phase reactions, under a low humidity environment (0–30%) and high solar radiation, gas-phase reactions are considered to play a key role in sulfate formation, while aqueous-phase formation reactions are more pronounced in a high humidity and weak solar radiation environment [54]. It is considered that levels of ambient SO_4_^2−^ in marine environments are related to anthropogenic emissions such as industry and shipping emissions [31,55,56].

For ambient NO_3_^−^ the primary formation pathway is oxidation of nitrogen oxides (NOx) to HNO_3_, and then the gaseous HNO_3_ reacts with NH_3_. Under ammonium-poor conditions and during nighttime the hydrolysis of N_2_O_5_ on the wet particles or droplets is also an important pathway that could generate particulate NO_3_^−^ [57]. The primary natural sources of NO_x_ are considered to be lightning, wildfires, soil microbial nitrogen cycle and stratospheric transmission, while anthropogenic sources of NO_x_ are mainly fossil fuel combustion, biomass combustion, agricultural activities, vehicle exhaust and human waste [58]. In marine environments, NO_3_^−^ may be partially present as NaNO_3_ due to the partition reactions between HNO_3_ and sea salt particles which mainly affects coarse particles (PM_10_) rather than PM_2.5_ [59].

Ambient NH_4_^+^ is produced from heterogeneous reactions of gas NH_3_ which is scavenged from air by water or gas to particle conversion reactions. NH_3_ is mainly produced from fertilizers, animal waste, human activity, industrial facilities, and wastewater treatments.

Traffic has been recognized as one of the possible air pollution sources with the approaching tourist season on the middle Adriatic coast in the previous study by Jakovljević et al. [38], carried out at the same location. The observed slightly higher contribution of SO_4_^2−^ to the bulk deposition mass during the warm period found in this study could be related with the combustion of sulphur-rich fuels and the presence of intensive shipping emissions. Furthermore, during the spring–summer period Cvitešić Kušan et al. [41] found a higher contribution of sulfates in PM_2.5_ originating from biological sources possibly related to the well-known increased phytoplankton production in the Adriatic due to favorable conditions, i.e., the availability of sunlight and nutrients within the sea surface layers [60]. The dimethyl sulfide (DMS), produced by biological activity in seawater, is the principal gaseous form of sulfur released to the air from the sea’s surface.

In addition, photochemical processing could also enhance the formation of secondary SO_4_^2−^ via oxidation by OH radicals. During warm periods, a higher contribution of NO_3_^−^ to bulk deposition mass was observed although we expected the lower values because the bulk samples are not refrigerated during sampling. These could be a consequence of a combined influence of several factors. Meteorological parameters (ambient temperature (T) and relative humidity (RH)) together with larger concentrations of precursor gases (NOx) because of higher traffic and higher amounts of atmospheric oxidants, such as O_3_, due to higher solar radiation, the surface property of pre-existing aerosols and its pH can influence and enhance the particulate NO_3_^−^ formation through gas to particle reactions. 

### 3.2. Major Ions in Wet Deposition

The summary statistics results of ion measurements in wet deposition samples are presented in Table 2, calculated as deposition fluxes in mass concentrations (mg m^−2^ d^−1^) and equivalent concentrations in μeq L^−1^.

Deposition fluxes of anions in wet deposition ranged from 0.41 mg m^−2^ d^−1^ to 7.0 mg m^−2^ d^−1^, while for cations from 0.04 mg m^−2^ d^−1^ to 4.92 mg m^−2^ d^−1^ (Table 2). The deposition fluxes of anions and cations in wet deposition were lower than those obtained in bulk deposition, which was expected, since bulk samples included both wet and dry deposition. However, equivalent concentrations of NO_3_^−^, SO_4_^2−^ and NH_4_^+^ were slightly higher in wet deposition. This could be explained by the higher volatility of NH_4_NO_3_, which had an impact on bulk deposition but not on wet deposition samples, as those were kept cold during the entire sampling. The average deposition fluxes of the determined anions and cations in wet deposition (in mg m^−2^ d^−1^) during the overall period of sampling followed the order NO_3_^−^ > Cl^−^ > SO_4_^2−^ > Na^+^ > Ca^2+^ > NH_4_^+^ > Mg^2+^ > K^+^. Compared to the order obtained for the overall bulk deposition, some differences are evident, for example in the higher abundance of SO_4_^2−^. The highest temporal variations in wet deposition flux among cations were found for Na^+^ and among anions for Cl^−^.

**Figure 5:**
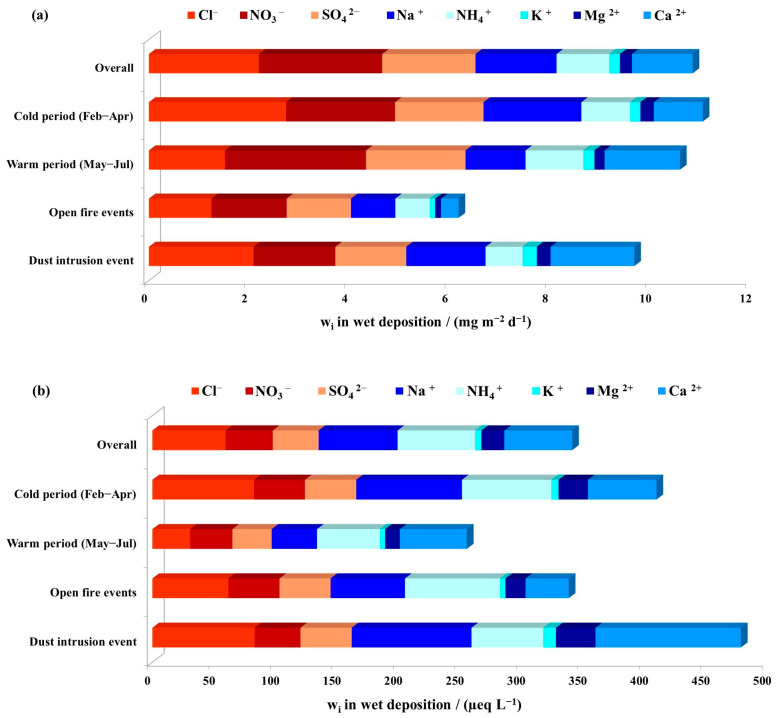
Average (**a**) deposition fluxes and (**b**) equivalent concentrations of ions in wet deposition during the sampling campaign from February to July 2019 at the middle Adriatic coastal site.

During the entire sampling period, the determined equivalent ion concentrations in wet deposition ranged from 2.37 μeq L^−1^ to 129.01 μeq L^−1^, while the average equivalent concentrations of ions in wet deposition (in μeq L^−1^) were very similar to those in bulk deposition and in the order Na^+^ > NH_4_^+^ > Cl^−^ > Ca^2+^ > NO_3_^−^ > SO_4_^2−^ > Mg^2+^ > K^+^ (Figure 5b). Anatolaki and Tsitouridou [61] reported a similar order, considered specific to urban environments in the Mediterranean and other arid regions. Maximum anion equivalent concentrations (for Cl^−^, NO_3_^−^ and SO_4_^2−^) in wet deposition were found in the cold period between 6 and 20 February and for Na^+^ between 5 and 20 March. The maximum equivalent concentrations for K^+^, Mg^2+^ and Ca^2+^ were observed in the sample collected during the dust intrusion event. Our results were lower in comparison with previously reported ion concentrations in wet deposition by Izquierdo and Avila [11] at a rural background site in northeastern Spain or by Keresztesi et al. [8] at four different sampling sites in the northern part of the inner eastern Carpathians in Romania. Our concentrations for Ca^2+^, NO_3_^−^ and NH_4_^+^ in wet deposition were higher than levels reported at Hawaii but lower compared to levels reported for Bermuda and the Philippines, as well as urban sampling sites on the east and west coasts of the US [62].

Seasonal differences of average ion fluxes and concentrations in wet deposition between the cold and warm period were observed (Figure 5), with higher values during the cold period. A statistically significant difference between average concentrations in the cold and warm period was observed only for Cl^−^, Na^+^ and Mg^2+^. During the cold period, average concentrations (in μeq L^−1^) followed the order Na^+^ > Cl^−^ > NH_4_^+^ > Ca^2+^ > SO_4_^2−^ > NO_3_^−^ > Mg^2+^ > K^+^, while during the warm period the order was Ca^2+^ > NH_4_^+^ > Na^+^ > NO_3_^−^ > SO_4_^2−^ > Cl^−^ > Mg^2+^ > K^+^. Higher deposition fluxes (in mg m^−2^ d^−1^) of Ca^2+^, Mg^2+^ and K^+^ were found during the dust intrusion event (Figure 5). A similar finding was presented in a study by Calvo et al. [33], which reported an about 3 times higher cation load, especially for Ca^2+^, Mg^2+^ and K^+^, at a rural station in Spain when air masses arrived from north Africa.

The average contributions of each anion to the total anion flux and each cation to the total cation flux in the wet deposition samples, calculated by mass concentrations, are presented on SI Figure S5 of Supplementary Information. The results are very similar to those reported for bulk samples except for NH_4_^+^ and K^+^. The average contributions of NH_4_^+^ to the total cation load were higher in the wet deposition samples, probably as a consequence of the wet sampling approach that prevented the evaporation of volatile ammonia compounds. 

### 3.3. Acidity and Neutralization Capacity of Atmospheric Deposition

Precipitation in unpolluted atmospheres has a slightly acidic pH (5.6), because naturally present carbon dioxide dissolves in cloud droplets and forms carbonic acid, a weak acid that can further ionize in water forming low concentrations of carbonate and hydronium ions [13,62,63]. Moreover, other acidic compounds naturally present in air, such as hydrogen sulfide and sulfur dioxide from volcanic eruptions or dimethyl sulfide in sea spray and nitrogen oxides from lightning, can also influence the pH of rainwater [63]. Therefore, precipitation with a pH lower than 5 is considered “acid rain” [64]. High emissions of SO_2_ and NO_x_, from local primary and secondary sources in the highly polluted areas are oxidized, forming sulfuric and nitric acid and further lower the precipitation acidity [65]. Furthermore, some special events such as dust intrusions can suppress precipitation acidity due to the high crustal alkaline minerals content, especially K^+^, Mg^2+^ and Ca^2+^ in the form of carbonates and bicarbonates [8,62]. Highly reactive gaseous ammonia (NH_3_) contribute to the precipitation acidity neutralization forming highly soluble ammonium salts (NH_4_^+^) [66]. Acidity can change the surface of particulate matter and thus affect its interactions with gas phase pollutants which together comprise atmospheric deposition. The acidity of atmospheric deposition mostly depends on the balance between the most abundant acidic ions (SO_4_^2−^, NO_3_^−^), neutralizing cations (Na^+^, NH_4_^+^, Ca^2+^, K^+^, Mg^2+^) and the main buffering agent carbonate/bicarbonate in its content, while Cl^−^ has much smaller influence, because of its higher ability to partition to gas-phase [67]. Understanding the balance and interactions between ions is crucial for predicting the possible influence of atmospheric deposition on the environment, especially on the bioavailability of some other elements.

To estimate the acidity of atmospheric deposition and acidification/neutralization capacity of ionic species, several approaches were applied and described in detail in Section S1 of Supplementary Information.

#### 3.3.1. Measurements of Bulk and Wet Deposition pH

**Figure 6:**
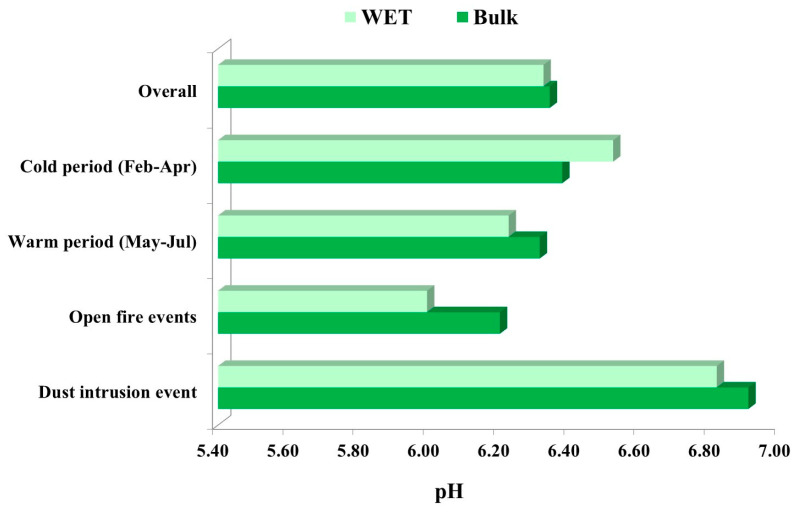
Average pH of bulk and wet deposition during the sampling campaign from February to July 2019 at the middle Adriatic coastal site.

Values for the obtained average pH during cold and warm periods, as well as during special events are presented in Figure 6. The measured pH of bulk and wet deposition samples ranged 5.7–6.9 and 5.8–6.8, respectively.

The average pH of bulk and wet atmospheric deposition for the entire 6-month sampling period was 6.3, which is much less acidic than natural rain and also higher than reported in some similar studies in Africa, America and Asia [28,62,65,68,69,70] and similar to the results obtained at background stations in Spain and Romania [8,33] and Sicily [71]. The obtained results are much less acidic than natural rain which is considered acid at pH 5.6 because of CO_2_ dissociation. The obtained results suggest a low Σanion-to-Σcation ratio in atmospheric deposition samples because of the high content of neutralizing species (probably NH_3_ or Ca^2+^) as well as the partial dissociation of acidic species. The average bulk and wet deposition pH levels were slightly lower during the warm period than during the cold period. This could have occurred because of higher humidity during the cold period (higher content of aerosol water) and more pronounced aqueous phase reactions. During the desert dust intrusion, bulk and wet deposition pH significantly increased, in comparison with the overall average pH. This was not surprising since in the same period higher loads of alkaline cations such as Ca^2+^, Na^+^ and Mg^2+^ were observed in both bulk and wet deposition (Figure 3 and Figure 5). Similar results were reported by [33]. These crustal alkaline cations in aeolian dust content affect the ion balance by influencing the phase partitioning of nitrate and ammonium in arid regions [72]. Since near the Martinska site most of soil is constituted from carbonate rocks (calcite, aragonite and dolomite), these crustal alkaline cations could also have local origin. 

The lowest pH levels in both bulk and wet deposition were measured during open-fire events (6.2 and 6.0, respectively). The relationship between pH and the equivalent concentrations of SO_4_^2−^ and NO_3_^−^ in bulk and wet deposition was examined. The obtained low correlation coefficient indicates that perhaps these species are not the main compounds that cause the acidity of atmospheric deposition at Martinska. Low pH levels during open-fire events may indicate higher (increased) emissions of other acidic compounds that contribute to the overall acidity of atmospheric deposition, most likely as organic acids since they are an important constituent of biomass burning emissions [62]. Moreover, the low pH could be explained by the presence of NH_3_ which can be used as a tracer for biomass burning emission and act in heterogeneous reactions as a weak base or be oxidized to form nitric acid (HNO_3_) which can further decrease pH.

The relationship between pH and the equivalent concentrations (in µeq L^−1^) of the most abundant anions and cations in bulk and wet deposition was examined. A high correlation coefficient was obtained for Ca^2+^, Mg^2+^ and Na^+^ in both bulk and wet deposition (SI Figures S6 and S7 of Supplementary Information) which suggests that these cations are the main factors that buffer the deposition’s acidity. Similar results were found by Anatolaki and Tsitouridou [61] in Greece and Roy et al. [73] in India.

#### 3.3.2. Acidity and Neutralization Capacity of Bulk and Wet Deposition

Fractional acidity (FA) was calculated to assess the level of acid neutralization in atmospheric deposition samples according to the equation given by Balasubramanian et al. [74] and Mishra et al. [16] (Section S1 of SI). The [H^+^]/[NO_3_^−^ + SO_4_^2−^] ratio for the overall period was between 0.002 and 0.04 in bulk deposition and between 0.002 and 0.03 in wet deposition. Higher FA values were previously reported at three rural stations in France by Pascaud et al. [75] (from 0.03 to 0.29, with an average of 0.17), at a coastal station in northern China by Xing et al. [65] (0.13) and at two urban sites in the northern part of India by Mishra and Anshumali [16] (0.01 and 0.09). The obtained FA values indicated that acidic anions (NO_3_^−^, SO_4_^2−^) in AD were neutralized by alkaline substances from 96% to 99.8%, which is in good agreement with the results obtained by Mishra and Anshumali [16] (99% and 91%), while in the previously mentioned studies by Pascaud et al. [75] and Xing et al. [65], AD was neutralized to about 80%.

The relative contribution of acidic species (SO_4_^2−^ and NO_3_^−^) to the total acidity in bulk and wet deposition at Martinska station, for the overall period, cold and warm sampling period, as well as during the open-fire and desert dust events was examined (SI Figure S8). Results show that in the bulk deposition, the contribution of NO_3_^−^ to the acidification ranged from 35.2% to 64.5% and for SO_4_^2−^ from 35.5% to 64.8%. In wet deposition, NO_3_^−^ contribution ranged from 45.1% to 58.2%, while SO_4_^2−^ contribution ranged from 41.8% to 54.9%. The average contribution of NO_3_^−^ to the acidification was estimated to be 53.1% and 50.9% for bulk and wet deposition, respectively. During the overall period, the average contribution for SO_4_^2−^ to the acidification of bulk and wet deposition was estimated to be 46.9% and 49.1%, respectively. The similar contribution for SO_4_^2−^ and NO_3_^−^ to the total acidity in bulk and wet deposition was probably the consequence of a complete dissociation HNO_3(g)_ and H_2_SO_4(g)_ in atmospheric deposition and content of low base (NH_3_, HCO_3_^−^), which can buffer dissociation reactions. Anatolaki and Tsitouridou [61] reported that in the wet deposition at a coastal station situated at the head of the Thermaikos Gulf in the north Aegean Sea the relative contribution was 67% and 33% for SO_4_^2−^ and NO_3_^−^, respectively. During both seasons and during open fire events, NO_3_^−^ had a higher contribution to the bulk and wet acidification and the only exception was the period with a desert dust intrusion when a higher contribution was obtained for SO_4_^2−^ probably because of the aerosol ageing process. The higher contribution of NO_3_^−^ to the acidity in bulk and wet deposition was attributed to the intensity of sources that contribute to overall pollution in Martinska. Sources as traffic and biomass burning both contributed to elevated levels of the NO_3_^−^ since they generate its gaseous precursors NOx. These sources could also generate SO_4_^2−^ gaseous precursors. Both NO_3_^−^ and SO_4_^2−^ in atmosphere are produced through gas phase and aqueous phase reactions which rate is dependent on ambient temperature, humidity, solar radiation, as well as presence of other oxidizing compounds. The gas phase production rate of NO_3_^−^ is faster in comparison with aqueous phase reactions which are slower and more pronounced for SO_4_^2−^ formation in high humidity and weak solar radiation environment. Moreover, sea-salt particles present in marine environment can serve as sites of limited sulphate production [13].

Neutralization factors (NF) of cations measured in bulk and wet deposition at Martinska site were calculated according to the Equation (S1) in Section S1 of SI in order to estimate its neutralization ability. The non-sea salt SO_4_^2−^ fraction (nssSO_4_^2−^) fraction was used in NF calculations because we assumed that the sea salt SO_4_^2−^ fraction (ss SO_4_^2−^) was buffered by the alkalinity of seawater. Table 3 shows the summary statistics of the obtained NF for the overall period. The NF of the neutralizing cations ranged 0.03–2.91 and 0.03–1.94 for bulk and wet deposition, respectively. In bulk deposition, the highest average neutralization factor for the overall period was obtained for Ca^2+^, followed by Na^+^ and NH_4_^+^. The same cations had the highest NF in wet deposition, but with a different order; Na^+^ > NH_4_^+^ > Ca^2+^. Lower NF values for Ca^2+^, NH_4_^+^ and Mg^2+^ were reported for the urban [28,76] and rural site [73], while a higher NF for Mg^2+^ was reported at the background site [76]. A higher NF for NH_4_^+^ was previously reported for some other locations in the northern part of the inner eastern Carpathians [8]. However, relatively lower NF_NH4_^+^ values obtained in bulk deposition, in comparison with wet, could be explained by its volatility, especially during summer months. Average NF_Mg_^2+^ and NF_K_^+^ were much lower compared to NF_Na_^+^ and NF_Ca_^2+^ obtained in both bulk and wet deposition. The obtained NF values for Ca^2+^, K^+^ and NH_4_^+^ in wet deposition were higher than previously reported by Roy et al. [73] at a rural station in India. The relative proportions of NF values for the major cations and for each sampling period are presented in the form of triangular diagrams (SI Figure S9).

The results show that a dust intrusion event significantly increased the NF values for Ca^2+^, Mg^2+^, K^+^ and Na^+^ in both bulk and wet deposition. During open-fire events only the levels of K^+^ in bulk deposition and NH_4_^+^ in wet deposition were higher than overall. The NFs obtained during cold period were higher than during the warm period. On average, Ca^2+^, Na^+^ and NH_4_^+^ were the predominant neutralizers in bulk deposition at the middle Adriatic region contributing about 43.7%, 34.2% and 22.2% toward the neutralization of bulk deposition and 30.7%, 35.6%, and 33.7% in wet deposition. Li et al. [28] reported higher contributions for Ca^2+^ and similar values for NH_4_^+^ in wet deposition at urban and rural sites in China. 

**Figure 7:**
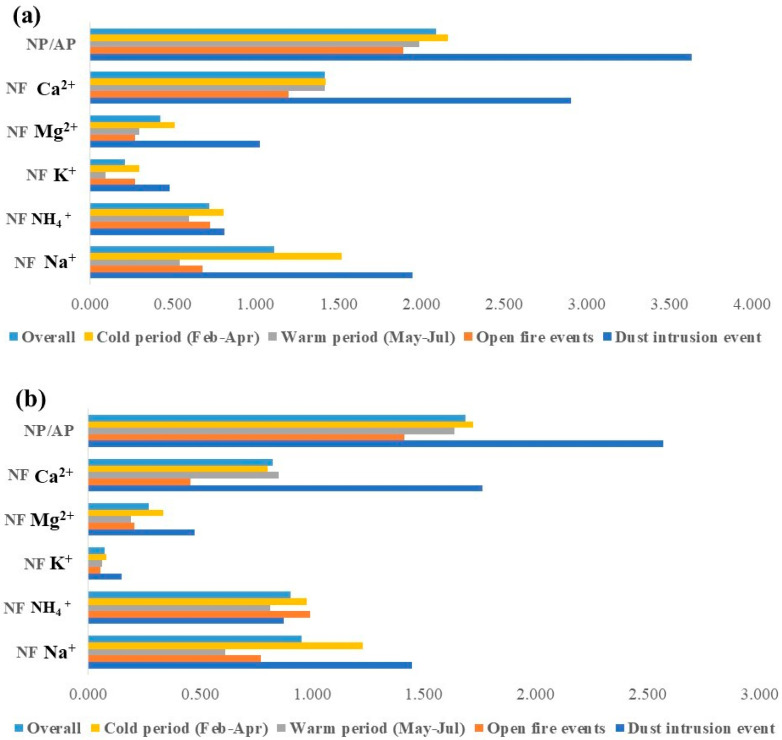
The average ratio of ionic neutralization potential (NP) to the acidification potential (AP) and average neutralization factors (NF) of individual cations in (**a**) bulk and (**b**) wet deposition during cold and warm seasons and special events.

In order to assess the balance between acidity and alkalinity in bulk and wet deposition at Martinska, the evaluated ratio between the neutralizing potential (NP) and the acidifying potential (AP) was calculated according to the Equation (S3) in Section S1 of SI. During the overall period as well as during special events, the NP/AP ratio was higher than 1 which indicates that the overall dominance of alkaline ionic species prevents the acidity of the bulk deposition (Figure 7). It was higher during the cold period compared to the warm period in both bulk and wet deposition. The NP/AP ratio was lowest during open-fire events, while during the dust intrusion, about twice higher values were obtained. The high NP/AP ratio suggests strong neutralization effects by inorganic cations (especially for Ca^+^, Na^+^, NH_4_^+^) offsetting the acidity caused by high loadings of acidic inorganic and organic anions which both contribute to the deposition acidity. 

**Figure 8:**
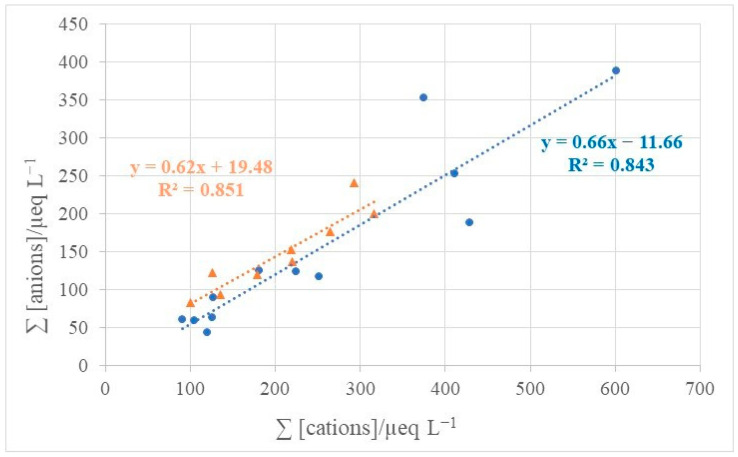
Ion balance of bulk (●) and wet (▲) deposition, expressed as Σanions-to-Σcations equivalent ratio.

Acid–base relations between the determined ionic compounds in atmospheric deposition were also examined through the ion balance calculation. Linear regression was performed between the sum of anion equivalents and the sum of cation equivalents for bulk and wet deposition. In addition to the measured anions, HCO_3_^−^ equivalent concentrations were calculated according to the Equation (S2) in Section S1 of SI using measured pH values. This correction did not significantly change the slope of the regression line: for bulk deposition it decreased by less than 3%, while for wet it increased by about 9%. Figure 8 presents Σanions-to-Σcations equivalent ratio with included calculated HCO_3_^−^ value. Both slopes in Figure 8 were lower than 1 (0.66 for bulk and 0.62 for wet deposition), which indicated acidic deposition properties, more pronounced for wet deposition. However, the obtained low Σanions-to-Σcations ratio also suggests that some other anions present in atmospheric deposition at Martinska were not measured, including anions of organic acids such as oxalic acid and methanesulphonic acid as well as inorganic phosphate, which are often abundant in marine atmospheres [41,67,77].

Multiple linear regression (MLR) is a statistical tool that can be used to analyze the relationship between one dependent variable and several independent variables in order to see which independent variable is the best predictor of the dependent variable. Unlike simple (univariate) regression, MLR is sensitive to combinations of variables, i.e., whether a predictor is important or not depends on the other predictors in the set, so the obtained significances may differ from simple regression results. Following the procedure explained in Anatolaki and Tsitouridou [61], we applied MLR to see which cations are the most important in explaining the neutralization of main anions, using the following equation:

[A^−^] = β_o_ + β_Na_[Na^+^] + β_NH4_[NH_4_^+^] + β_K_[K^+^] + β_Mg_[Mg^2+^] + β_Ca_[Ca^2+^] (1)

where the dependent variable [A^−^] is the equivalent concentration of the anion, β_i_ corresponding correlation coefficient of cation and β_o_ the intercept. Alternatively, the relationship between [A^−^] and cations can be expressed using standardized regression coefficients β*_i_ by equation [A^−^] = β*_Na_[Na^+^] + β*_NH4_[NH_4_^+^] + β*_K_[K^+^] + β*_Mg_[Mg^2+^] + β*_Ca_[Ca^2+^]. Standardized regression coefficients enable us to see which independent variable has the greatest effect on the dependent variable. However, the magnitude of the coefficient does not provide information on the importance of the predictor variable in explaining the variation in the dependent variable. Due to the relatively small number of samples, we included all the measured cations in the equation, except [H^+^]. The results are presented in Table 4.

The results in Table 4 show that the equivalent concentrations of five measured cations explain between 96.3% and 97.6% of the anion variability in bulk deposition, and between 90.8% and 99.3% of anion variability in wet deposition. However, in the case of SO_4_^2−^ none of the variables were statistically significant. For NO_3_^−^, the major neutralizing cation was NH_4_^+^, in both bulk and wet deposition. K^+^ was in significant negative correlation only in bulk deposition, which was probably more related to the influence of different sources (such as biomass burning) than neutralization. For Cl^−^ in bulk deposition, the only significant neutralization cation was Na^+^, which is related with the marine character of the location. In wet deposition, Cl^−^ correlated negatively with Ca^2+^, while the only significant neutralization cation was Mg^2+^ (marine source). In addition to the NF-approach, which identified cations Ca^2+^, NH_4_^+^ and Na^+^ as the most important neutralizers, MLR allowed us to see to which extent is the neutralization capacity of an individual cation related to the specific ion. Previous studies that used MLR for neutralization capacity assessment [61,67,78] mostly found that Ca^2+^ and NH_4_^+^ were the main neutralizing cations for SO_4_^2−^ and NO_3_^−^. Meng et al. [67] in rainwater samples collected in Shanghai also found that the neutralizing effect of NH_4_^+^ on SO_4_^2−^ and NO_3_^−^ was higher than that of Ca^2+^; standardized regression coefficients for NH_4_^+^ were 0.459 and 0.483 for SO_4_^2−^ and NO_3_^−^, respectively, while for Ca^2+^ they were 0.258 and 0.227. In this study, considering the neutralization of nitrate, the highest standardized regression coefficients were also obtained for NH_4_^+^; they were 1.915 and 0.907 for bulk and wet deposition, respectively. For the neutralization of sulphate in bulk deposition, the greatest effect also had NH_4_^+^ (0.746) while in wet had Mg^2+^ (3.312). Our results are more similar with the study carried out in Anatolia, where Ca^+2^ and NH_4_^+^ were found to be the main neutralizers, but MLR also revealed an important participation of Mg^2+^ and Na^+^ in the neutralization process [78], with significant positive Mg^2+^ regression coefficients (0.22 and 0,24 for SO_4_^2−^ and NO_3_^−^, respectively) and positive Na^+^ regression coefficients (0.13 and −0.15 for SO_4_^2−^ and NO_3_^−^. respectively). Gillet at al. [79], analysing the rainwater samples collected in Jabiru, Australia, also found that ammonium was significant as a counter-ion for the anions, while sodium was significant variable only for chloride, with regression coefficient 0.63- Calcium was not a significant variable and was excluded from equations. The multiple regression equations with corresponding regression coefficients obtained in this and similar studies are presented in detail in Table S2 of the Supplementary Information.

In conclusion, data analysis for the Martinska site showed that the highest influence on the overall acidity of both bulk and wet deposition came from NO_3_^−^ while SO_4_^2−^ has the highest influence only during dust intrusions. The highest neutralization ability among the measured cations came from Ca^2+^, Na^+^ and NH_4_^+^. The relatively low ion balance ratio also suggested that certain other acidic organic anions are probably present in atmospheric deposition.

### 3.4. Dominant Sources of Ions in Atmospheric Deposition

#### 3.4.1. Correlations between Inorganic Compounds in Atmospheric Deposition

The correlation coefficients of determined ionic species in bulk and wet deposition are presented in SI Table S3 of Supplementary information. In bulk deposition, most of the ions significantly correlated with each other, with the exception of Ca^2+^ and K^+^ with Cl^−^ and NO_3_^−^. The highest correlation was found between Na^+^ and Cl^−^ (r = 0.966), which is not surprising since Martinska is a coastal location. High correlations were also found between SO_4_^2−^ and Na^+^ as well as between Mg^2+^ and Na^+^, which indicated natural origin (sea spray), while the correlation between SO_4_^2−^ and NH_4_^+^ could have been due to the formation of secondary aerosols, from precursors of probably anthropogenic origin. During aerosol aging, gaseous NH_3_ reacts with gaseous acids H_2_SO_4_ and HNO_3_ and forms particulate salts (NH_4_)_2_SO_4_ and NH_4_NO_3_ [67].

Significant correlations between NH_4_^+^ and K^+^ in both bulk and wet deposition indicate biomass burning and/or agricultural activity as the sources present in the vicinity of Martinska, while the significant correlation between NO_3_^−^ and SO_4_^2−^ suggested similar chemical behaviors or/and a common source of their gaseous precursors (SO_2_ and NO_x_) as the combustion of fossil fuels.

In wet deposition, only a few statistically significant correlations were found. Similarly to bulk deposition, a very strong correlation was found between Na^+^ and Cl^−^, Na^+^ and SO_4_^2−^, Mg^2+^ and Cl^−^, and Mg^2+^ and Na^+^ pointing to marine sources. Contrary to bulk deposition, no correlation between Ca^2+^ and Mg^2+^ was found (crustal source). NO_3_^−^ did not significantly correlate with any other ions. Ma et al. [62] explained the high correlation between K^+^ and SO_4_^2−^ and Cl^−^ in wet depositions through the existence (presence) of K_2_SO_4_ salts and aged smoke, since KCl can react with sulphate carrying compounds produced in fire emissions.

#### 3.4.2. Estimation of Marine and Non-Marine Contribution

The chemical composition of precipitation in the middle Adriatic-sub basin is under the synergistic influence of local anthropogenic and natural sources as well as under the influence of long-range transport of highly enriched air masses from southern and northern countries [26,41,80]. One of the receptor models in source apportionment used to determine whether ions are of marine or crustal origin is the enrichment factor (EF) calculation. In general, the content of ions in atmospheric deposition is considered to be highly enriched if EF > 100, moderately enriched if 10 < EF < 100, less enriched if EF < 10 or no-enriched if EF < 1. From the obtained equivalent ratio between ions in deposition, in comparison with a theoretical ratio of ion and a reference element, it can be derived whether the ions in precipitation have marine, EF_(Xi)marine_ (Equation (5)), or crustal, EF_(Xi)crustal_ (Equation (6)) origin. The Martinska measuring site is situated on the coast, so the hypothesis that all sodium in the deposition originated from sea salt (ss) was applied. In previous research [8,76,81] Na^+^ was used as a reference element for the calculation of the ss fraction.

EF_(Xi)marine_ = (X_i_/Na^+^)_sample_/(X_i_/Na^+^)_marine_ (2)

EF_(Xi)crustal_ = (X_i_/Ca^2+^)_sample_/(X_i_/Ca^2+^)_crustal_ (3)

Values of the theoretical equivalent ratio of each ion and Na^+^ in seawater were given by Keene et al. [81] and Chen et al. [76]: [SO_4_^2−^/Na^+^]_seawater_ = 0.121; [Cl^−^/Na^+^]_seawater_ = 1.161; [Ca^2+^/Na^+^]_seawater_ = 0.044; [Mg^2+^/Na^+^]_seawater_ = 0.227; [K^+^/Na^+^]_seawater_ = 0.022.

The terrigenous (crustal) contribution of K^+^, Mg^2+^ and SO_4_^2−^ in atmospheric deposition samples can be estimated by applying the specific equivalent ratios given by Keresztesi et al. [8]: [SO_4_^2−^/Ca^2+^]_crustal_ = 0.0188; [Ca^2+^/Mg^2+^]_crustal_ = 1.78; [K^+^/Ca^2+^]_crustal_ = 0.504; [Cl^−^/Ca^2+^]_crustal_ = 0.0031; [Na^+^/Ca^2+^]_crustal_ = 0.569.

Calculated marine and crustal EF factors (calculated by μeq L^−1^) of the measured ions in bulk and wet deposition during overall sampling period are presented in SI Tables S4 and S5.

The results indicate that the ions determined in bulk deposition at Martinska station have marine and crustal or only crustal i.e., lithogenic origin. EF_marine_ for Cl^−^ was below 1 which indicated that the content of Cl^−^ in bulk deposition was not enriched by contribution from other sources than marine. Furthermore, EF_crustal_ values for Cl^−^ were moderately to highly enriched which indicated that Cl^−^ in bulk deposition was present in crustal form as sea salt. EF_marine_ values for Mg^2+^, K^+^ and SO_4_^2−^ ions were less to moderately enriched, which suggests the contribution of both, marine and crustal i.e., lithogenic sources on bulk deposition content. Calculated EF_marine_ values for Ca^2+^ were moderately to highly enriched which suggests a higher contribution of lithogenic sources than marine sources on its bulk deposition content. The obtained EF_crustal_ values for Mg^2+^, K^+^, Na^+^ and SO_4_^2−^ were mostly below 1, indicating their crustal i.e., lithogenic origin.

**Figure 9:**
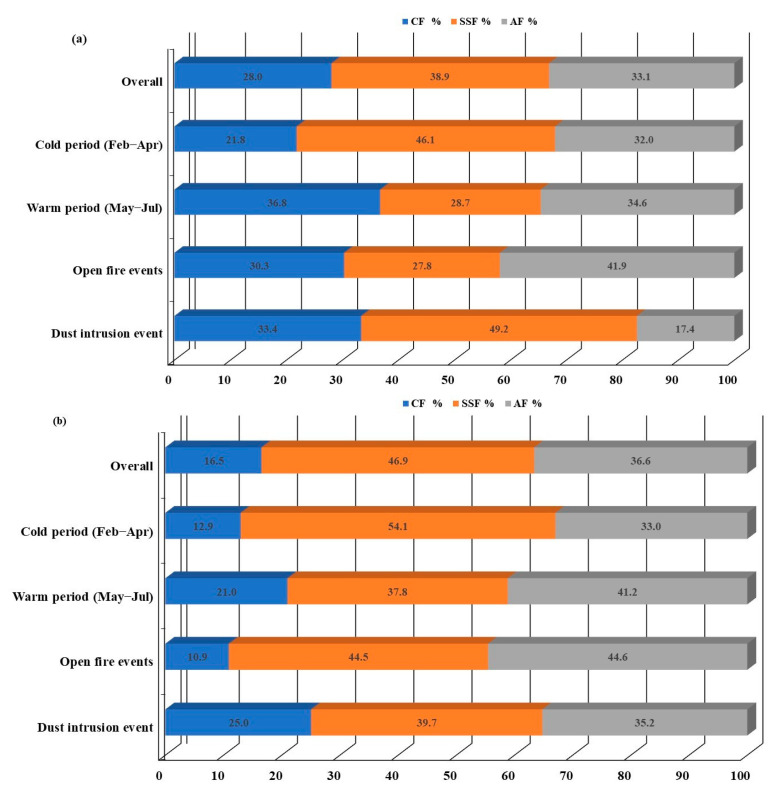
Determined contribution of crustal fraction (CF), sea-salt fraction (SSF) and anthropogenic fraction (AF) in (**a**) bulk and (**b**) wet deposition to the overall investigated period as well as during different seasons and specific events in the middle Adriatic region.

In order to estimate the contribution of marine and non-marine sources, the contributions of sea salt source fraction (SSF), crustal source fraction (CF) as well as anthropogenic source fraction (AF) were calculated according to Equations (4)–(6) given by Chen et al. [76], Keresztesi et al. [8] and Xing et al. [65] (Figure 9).

SSF(%) = (1/EF_marine_) × 100 (4)

CF(%) = (1/EF_crustal_) × 100 (5)

AF(%) = 100 − SSF − CF(6)

However, in this context it should be kept in mind that AF actually represents all other sources, which are not necessarily all anthropogenic. For example, this approach cannot separate anthropogenic biomass burning from natural biomass burning sources, and it also includes products of gas-to-particle conversion (i.e., secondary aerosols) as well as combustion derived material.

Results show that for the overall sampling period at Martinska station, the AF, SSF and CF sources contributed 33.1%, 38.9% and 28% to the bulk deposition, respectively. In wet deposition, contributions were 36.6%, 46.9% and 16.5% for AF, SSF and CF, respectively (Figure 9). Similar levels for AF, SSF and CF in wet deposition were reported at an urban and rural site by Keresztesi et al. [8], while Li et al. [28] reported higher levels. The obtained results could be explained by differences in sampling between bulk and wet deposition and/or are related to differences in the nature of removal mechanisms of pollutants present in the air at the Martinska site. Bulk deposition is mainly a pathway for removal of coarse pollutants of crustal origin, while wet deposition is mainly a pathway for removal of fine and gaseous pollutants of anthropogenic or marine origin. The precipitation amount can also influence the obtained differences. Results show that the estimated contribution for AF, SSF and CF in bulk and wet deposition during different seasons and special events differed slightly from overall average. In bulk deposition, the SSF contribution during cold period and dust intrusion event was higher than for the overall period (46.1% and 49.2%, respectively), while the AF and CF contribution was higher during the warm period and open-fire events than overall. During the warm period and open-fire events the AF contribution was 34.6% and 41.9%, respectively, while the CF contribution was 36.8% and 30.3%, respectively.

The AF contribution in wet deposition was lower than the overall average during the cold period but higher during the warm period and open-fire events, while during dust intrusion events the AF contribution did not differ significantly. During open-fire events and the warm period, the AF contribution was estimated to be 44.6% and 41.2%, respectively. In wet deposition, the contribution of CF was lower than in bulk, while the opposite was true for SSF. The contribution of the SSF fraction to wet deposition was higher than overall average only during the cold period, when it was estimated to be 54.1%. CF fraction contribution was slightly higher than the overall average during the warm period, while during dust intrusion events the contribution of the CF fraction was estimated to be 25%, an increase of about 9% compared to the overall period. The SSF enrichment in the cold period in both bulk and wet deposition samples at Martinska could be explained by meteorology. Frequent high-speed winds in the cold period at Martinska [20] can interact with the sea’s surface and increased emission of primary sea spray aerosols whose formation is favorable under lower air temperatures. The higher contribution of CF during the warm period could be explained by soil resuspension, which is also enhanced by greater human activity during the tourist season. Furthermore, Penezić et al. [26] showed that during the warm period, especially June and July, Martinska is under the influence of air masses from the south Mediterranean, which probably causes higher loads of ions that have crustal i.e., lithogenic origin in atmospheric deposition. The AF contribution increased during open-fire events, possibly due to intense emissions of particles and gases that contribute to formation of secondary aerosols and ultimately to the deposition amount of substances characterized as anthropogenic. Cvitešić Kušan et al. [41] explained similarly the elevated levels of fine particulate matter during fire events at the middle Adriatic coastal station.

## 4. Conclusions

Major ions (Cl^−^, NO_3_^−^, SO_4_^2−^, Na^+^, K^+^, NH_4_^+^, Mg^2+^, Ca^2+^) in bulk and wet deposition were measured simultaneously over a 6-month period at a coastal background location. The data were analyzed with respect to seasonal variations and special events such as dust intrusion and local open-fires, typical of Mediterranean coastal regions. To our best knowledge, this is the first study with parallel measurements of ionic content in both types of atmospheric deposition samples for the eastern middle Adriatic region.

The highest deposition fluxes and equivalent concentrations at the Martinska site had Cl^−^ in bulk and NO_3_^−^ in wet deposition. Among the cations, the highest levels were found for Ca^2+^, Na^+^ and NH_4_^+^ in both bulk and wet deposition. A clear seasonal difference was found between deposition fluxes and equivalent concentrations of ions determined in the cold and warm sampling periods. For all ions except Ca^2+^, higher average fluxes were determined during the cold period, especially for Cl^−^, Na^+^, Mg^2+^ and K^+^. The main reason could have been the increased formation of primary sea spray aerosols in the cold period due to influence of meteorological parameters. Dust intrusion caused a significant increase for most of the ions, especially Ca^2+^, Mg^2+^ and K^+^, while during open-fire events only the levels of K^+^ were higher in bulk deposition samples. The measured anions and cations contributed to the bulk deposition mass about 21.2% and 15.5%, respectively. Among the anions, the highest average percentage contribution came from NO_3_^−^ in both the bulk and wet deposition. Ca^2+^ had the highest contribution to the total cations in bulk deposition, while in wet deposition the highest contribution came from Na^+^ and NH_4_^+^.

The deposition acidity and neutralization capacity of major cations showed seasonal differences as well as the influence of special events, especially dust intrusion. The measured pH of AD was slightly acidic (5.7–6.9), with the highest values observed during the dust intrusion. Low ionic balance ratio indicated overall acidic deposition properties (more pronounced for wet deposition) and suggested that some other anions, which were not measured herein, were present in the atmospheric deposition. However, the ratio between neutralization and acidification potential indicated the dominance of alkaline ionic species preventing the acidity of deposition. The highest influence on the overall acidity of both bulk and wet deposition came from NO_3_^−^, while nssSO_4_^2−^ had the highest influence only during dust intrusions. The fractional acidity calculation indicated that major acidic anions, NO_3_^−^ and nssSO_4_^2−^, were neutralized by alkaline ionic species to between 96% and 99.8%. The highest neutralization ability among the measured cations was determined for Ca^2+^, Na^+^ and NH_4_^+^. For NO_3_^−^, NH_4_^+^ was found to be the major neutralizing cation in both bulk and wet deposition.

Regression analysis and enrichment factor calculations were applied to estimate possible sources. The results indicated several natural (marine, crustal) and anthropogenic sources of major ions in AD. The contributions of the sea salt source fraction and crustal source fraction differed in bulk and wet deposition. Wet deposition was characterized by a higher contribution of SSF (46.9%) compared to bulk deposition (38.9%) and lower contribution of CF. The anthropogenic source fraction was similar for both types of deposition samples (about 39%). The SSF contribution was higher than overall during the cold period and dust intrusion event, while the AF contribution was higher during warm period and open-fire events. The CF contribution was slightly higher in both bulk and wet deposition during the warm period, while during dust intrusion events its contribution increased by about 5% in bulk and 9% in wet deposition. For a better identification of sources, future studies should also include measurements of specific organic compounds.

Given the limited project budget and duration, and relatively small number of collected samples, we could not carry out a more sophisticated source apportionment analysis. However, the results of this research, in addition to providing the first data on the ionic composition of wet and bulk deposition in the eastern middle Adriatic region, also clearly point out some specific sources that significantly affect ion levels and the acid-base balance in the environment. Furthermore, more detailed studies of the events such as desert dust intrusion and forest fires are needed in the future, as the research through this project has shown that they significantly affect air quality and the composition of deposited matter. These findings are in accordance with the latest WHO guidelines [82], which also emphasize the importance of monitoring the contributions from natural sources. The impact of traffic on air pollution was also clearly recognized, which should be taken into account in developing sustainable tourism.

## Figures and Tables

**Table 1:** Summary statistics of determined bulk deposition fluxes, major ions fluxes and ion equivalent concentrations for the entire sampling period.

	Deposition Flux/mg m^−2^ d^−1^								
Statistical Parameter	Bulk Deposition	Cl^−^	NO_3_^−^	SO_4_^2−^	Na^+^	NH_4_^+^	K^+^	Mg^2+^	Ca^2+^
C_min_	22	0.15	3.04	1.39	0.20	0.65	0.11	0.12	1.70
C_max_	185	19.44	5.36	7.54	10.19	4.05	5.75	1.50	7.11
C_avg_	71	5.61	4.25	3.25	3.43	1.61	1.13	0.65	3.33
SD	50	6.32	0.78	1.79	3.46	0.89	1.63	0.52	1.63
concentration/µeq L^−1^									
C_min_		2.25	25.81	15.21	4.66	18.96	1.31	4.57	38.29
C_max_		265.07	48.71	88.54	249.69	126.88	83.12	64.41	173.79
C_avg_		82.09	35.30	35.28	78.43	46.57	15.14	28.14	85.46
SD		92.64	8.21	21.20	81.22	28.89	23.14	22.33	42.00

C_avg_–average value, C_min_, C_max_–minimum and maximum values, SD–standard deviation.

**Table 2:** Summary statistics of determined ion deposition fluxes and ion equivalent concentrations in wet deposition for the entire sampling period.

	Deposition Flux/mg m^−2^ d^−1^							
Statistical Parameter	Cl^−^	NO_3_^−^	SO_4_^2−^	Na^+^	NH_4_^+^	K^+^	Mg^2+^	Ca^2+^
C_min_	0.41	0.59	0.47	0.28	0.34	0.04	0.04	0.15
C_max_	7.00	4.38	3.28	4.92	1.99	0.45	0.66	2.71
C_avg_	2.19	2.46	1.86	1.62	1.06	0.21	0.24	1.21
SD	2.03	1.45	1.15	1.44	0.60	0.15	0.19	0.95
concentration/µeq L^−1^								
C_min_	21.41	27.58	19.79	22.95	28.00	2.37	7.21	16.10
C_max_	129.01	53.55	58.33	113.38	104.71	10.11	32.01	118.22
C_avg_	59.50	38.30	37.34	64.12	63.29	4.96	18.52	55.16
SD	37.34	8.77	10.08	33.62	26.20	2.42	9.60	31.38

C_avg_–average value, C_min_, C_max_–minimum and maximum values, SD–standard deviation.

**Table 3:** Neutralization factors of major cations ions in bulk and wet deposition for the overall sampling period.

		NF_Na_	NF_NH4_	NF_K_	NF_Mg_	NF_Ca_
Bulk Deposition	Average	1.11	0.72	0.21	0.42	1.42
	SD	0.95	0.20	0.25	0.29	0.60
	Min	0.12	0.47	0.03	0.11	0.72
	Max	2.82	1.19	0.78	1.02	2.91
Wet Deposition	Average	0.95	0.90	0.07	0.27	0.82
	SD	0.49	0.20	0.03	0.13	0.46
	Min	0.35	0.65	0.05	0.11	0.28
	Max	1.94	1.28	0.15	0.50	1.76

**Table 4:** MLR analysis for equivalent anion concentrations in bulk and wet deposition; statistically significant regression coefficients (*p* < 0.05) were marked with *.

	Anion	β_0_	β*_Na_ β_Na_	β*_NH4_ β_NH4_	β*_K_ β_K_	β*_Mg_ β_Mg_	β*_Ca_ β_Ca_	R^2^
Bulk Deposition	[NO_3_^−^]		−0.339	1.915 *	−1.225 *	0.353	−0.006	
		15.667 *	−0.034	0.544 *	−0.435 *	0.130	−0.001	0.976
	[SO_4_^2−^]		0.074	0.746	−0.195	0.449	−0.083	
		2.540	0.019	0.548	−0.178	0.427	−0.042	0.963
	[Cl^−^]		1.278 *	−0.375	0.063	−0.031	−0.067	
		36.155	1.457 *	−1.202	0.252	−0.131	−0.147	0.973
Wet Deposition			−0.342	0.907 *	−0.045	0.536	−0.049	
	[NO_3_^−^]	17.288 *	−0.089	0.304 *	−0.163	0.489	−0.014	0.966
	[SO_4_^2−^]		−2.598	0.409	0.608	3.312	−1.297	
		23.397 *	−0.779	0.157	2.534	3.475	−0.417	0.908
	[Cl^−^]		−0.140	0.064	0.024	1.305 *	−0.522 *	
		2.188	−0.156	0.091	0.365	5.075 *	−0.621 *	0.993

## Data Availability

The data presented in this study are available on request.

## Supplementary Materials

The following supporting information can be downloaded at: https://www.mdpi.com/article/10.3390/toxics11070551/s1.
