# Supplementary material for: Sources, Ionic Composition and Acidic Properties of Bulk and Wet Atmospheric Deposition in the Eastern Middle Adriatic Region
Source: Toxics. 2023 Jun 23;11(7):551. doi: 10.3390/toxics11070551 (PMC10383331; doi:10.3390/toxics11070551)
Supplement: Supplementary file 1 [file toxics-11-00551-s001.zip › toxics-2361541-supplementary.pdf]

toxics

MDPI

Supplementary materials

Sources, Ionic Composition and Acidic Properties of Bulk and Wet Atmospheric Deposition in the Eastern Middle Adriatic Region

Valentina Gluščić [1], Silva Žužul [1,*], Gordana Pehnec [1], Ivana Jakovljević [1], Iva Šimić [1], Ranka Godec [1], Ivan Bešlić [1], Andrea Milinković [2], Saranda Bakija Alempijević [2] and Sanja Frka [2]

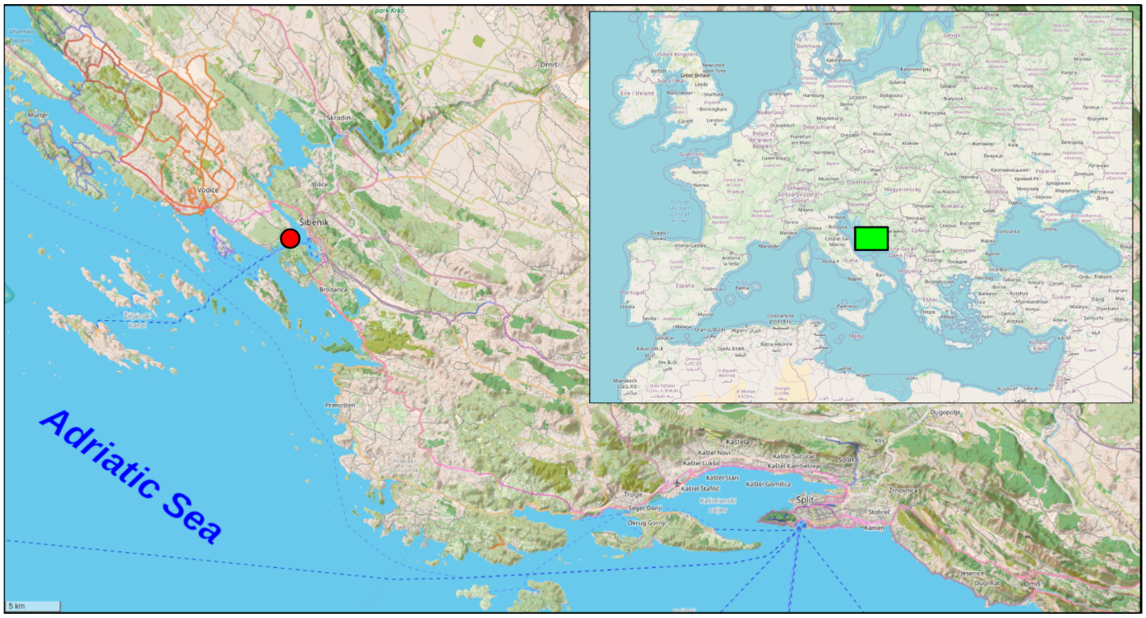

Figure S1. Sampling site Martinska (red dot) at the Middle Adriatic coast area (green square) (modified from https://www.openstreetmap.org).

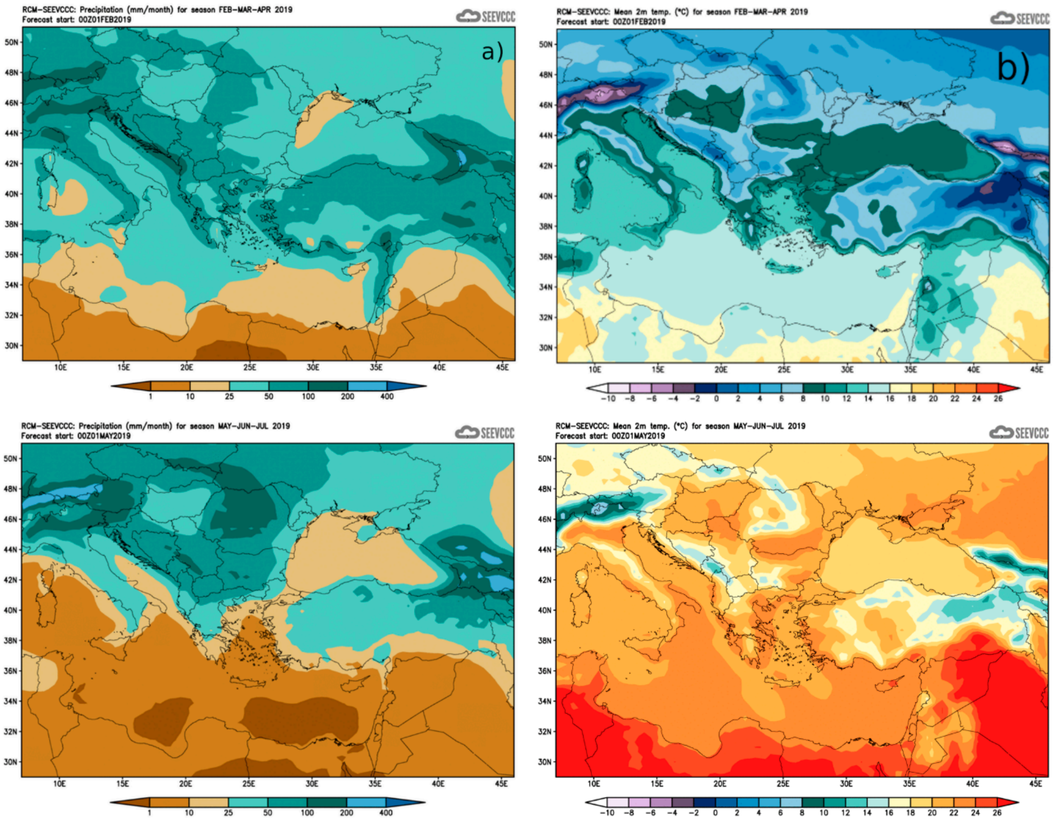

Figure S2. Meteorological parameters patterns in cold and warm season a) precipitation amount b) temperature (adopted from http://www.seevccc.rs, accessed on 5 April 2023).

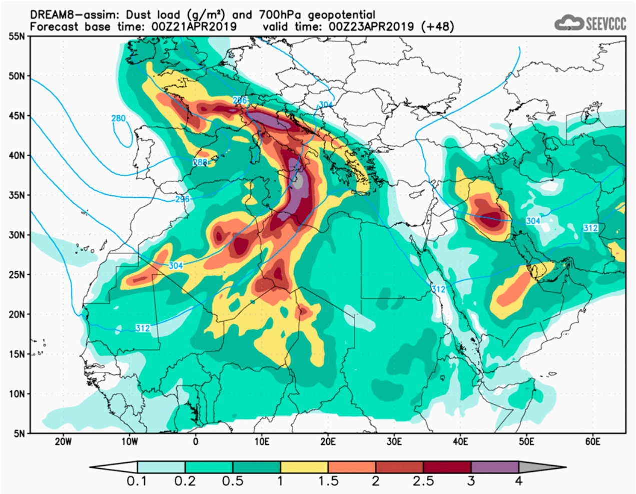

Figure S3. Dust loads from Sahara from 21st until 25th of April 2019 (adopted from http://www.seevccc.rs, accessed on 5 April 2023).

Table S1. Determined ion deposition fluxes (mg m^{-2} d^{-1}) and ion equivalent concentrations (μeq L^{-1}) in bulk and wet deposition content in comparison with literature (average mean values as well as minimum and maximum values in brackets) *only maximum values.

**Bulk deposition**										
Location	Type of measuring site	**Cl^- mg m^{-2} d^{-1}**	**NO_3^- mg m^{-2} d^{-1}**	**SO_4^{2-} mg m^{-2} d^{-1}**	**Na^+ mg m^{-2} d^{-1}**	**NH_4^+ mg m^{-2} d^{-1}**	**K^+ mg m^{-2} d^{-1}**	**Mg^{2+} mg m^{-2} d^{-1}**	**Ca^{2+} mg m^{-2} d^{-1}**	Reference
the Eastern Middle Adriatic coast	a coastal-rural measuring site	5.61 (0.15- 19.44)	4.25 (3.04 – 5.36)	3.25 (1.39 – 7.54)	3.43 (0.2 – 10.19)	1.61 (0.65 – 4.05)	1.13 (0.11 – 5.75)	0.65 (0.12 – 1.50)	3.33 (1.70 – 7.11)	***This study***
East Mediterranean, Aliaga region, western part of Turkey at coast of Aegean Sea	industrial				5.968		1.960	5.794	28.168	Kara et al. 2014
	residential area				5.293		3.426	2.539	9.820	
	background				2.698		0.951	0.850	3.748	
the south centre part of Cuba on the coast of the Caribbean Sea	rural area				2.801 (1.027 - 8.008)		0.637 (0.232 – 1.085)	0.493 (0.205 – 1.247)	3.259 (1.692 – 8.051)	Morera-Gómez et al. 2019
Middle Mediterranean, North Adriatic, the Lagoon of Venice	industrial				2.684		0.978	1.130	6.782	Rossini et al. 2005
	urban				5.250		1.327	1.354	3.849	
	a remote site B				3.925		1.024	1.759	6.046	
	a remote site C				4.596		0.801	1.038	3.511	
Western Mediterranean, the Isle of Mallorca, Balearic Islands, Spain	regional background	20.93	4.03	7.47	10.42	0.57	1.69	1.34	2.91	Cerro et al. 2020
Location	Type of measuring site	**Cl^- μeq L^{-1}**	**NO_3^- μeq L^{-1}**	**SO_4^{2-} μeq L^{-1}**	**Na^+ μeq L^{-1}**	**NH_4^+ μeq L^{-1}**	**K^+ μeq L^{-1}**	**Mg^{2+} μeq L^{-1}**	**Ca^{2+} μeq L^{-1}**	
the Eastern Middle Adriatic coast	a coastal-rural measuring site	82.09 (2.25 – 265.07)	35.3 (25.81 - 48.71)	35.28 (15.21 – 88.54)	78.43 (4.66 – 249.69)	46.57 (18.96 – 126.88)	15.14 (1.31 – 83.12)	28.14 (4.57 – 64.41)	85.46 (38.29 – 173.79)	***This study***
Western Mediterranean, North East Spain	rural background site	16.3 (3.64 – 103)	35.5 (5.29 – 167)	27.7 (6.10 – 138)	14.7 (2.99 – 89.4)	27.8 (1.62 – 129)	4.14 (0.66 – 17.7)	10.1 (0.84 – 61.3)	71.7 (5.39 – 609)	Izquierdo and Avila 2012

Middle Mediterranean, Northern Italy, Po Valley	residential urban	206 (9.8 – 3487.7)	143.9 (10.1 – 1592.7)	265.3 (13.9 – 3336.5)	178.4 (5.4 – 3161.5)	111.2 (0 – 948)	57.3 (0-803.6)	55.2 (0 – 639.4)	279.3 (10.7 – 3016.0)	Pieri et al. 2010
	rural	115.1 (12.9 – 811.8)	165.4 (12.9 – 1909.1)	195.5 (18.2 – 1936.7)	115.1 (8.7 – 828.1)	148.1 (0 – 857.1)	32.9 (0-247.8)	35.5 (0 – 375.8)	206.3 (17.0 – 2095.8)	
Wet deposition										
Location	Type of measuring site	**Cl^- mg m^{-2} d^{-1}**	**NO_3^- mg m^{-2} d^{-1}**	**SO_4^{2-} mg m^{-2} d^{-1}**	**Na^+ mg m^{-2} d^{-1}**	**NH_4^+ mg m^{-2} d^{-1}**	**K^+ mg m^{-2} d^{-1}**	**Mg^{2+} mg m^{-2} d^{-1}**	**Ca^{2+} mg m^{-2} d^{-1}**	Reference
the Eastern Middle Adriatic coast		2.91 (0.41–7)	2.46 (0.59-4.38)	1.86 (0.47-3.28)	1.62 (0.28-4.92)	1.06 (0.34-1.99)	0.21 (0.04-0.45)	0.24 (0.04-0.66)	1.21 (0.15-2.71)	***This study***
Western Mediterranean, the Isle of Mallorca (Spain)	regional background	15.39 (206.614*)	3.13 (35.046*)	6.16 (100.492*)	7.75 (119.755*)	0.52 (6.907*)	1.03 (13.064*)	0.99 (12.966*)	1.68 (20.999*)	Cerro et al. 2020
Location	Type of measuring site	**Cl^- μeq L^{-1}**	**NO_3^- μeq L^{-1}**	**SO_4^{2-} μeq L^{-1}**	**Na^+ μeq L^{-1}**	**NH_4^+ μeq L^{-1}**	**K^+ μeq L^{-1}**	**Mg^{2+} μeq L^{-1}**	**Ca^{2+} μeq L^{-1}**	
the Eastern Middle Adriatic coast	a coastal-rural measuring site	59.50(21.41-129.01)	38.30(27.58-53.55)	37.34(19.79-58.33)	64.12(22.95-113.38)	63.29(28.00-104.71)	4.96(2.37-10.11)	18.52(7.21-32.01)	55.16(16.10-118.22)	***This study***
West Mediterranean, North East Spain	rural background site	10.8 (2.20-78.2)	33.5 (3.49-161)	26.1 (4.40-151)	11.0 (1.80-78.7)	40.2 (3.44-126)	3.27 (0.50-14.6)	7.22 (0.93-63.0)	56.6 (5.05-723)	Izquierdo and Avila 2012
Eastern Mediterranean, the city center of Thessaloniki, northern Greece	Urban site	70.3(9.0-827)	72.7(15.2-498)	163(26-1223)	73.3(11.7-473)	56.1(0.87-781)	15.3(0.51-126)	31.9(2.33-562)	323(10.5-1788)	Anatolaki and Tsitouridou 2009
Eastern Mediterranean, the city center of Thessaloniki, northern Greece	Urban site	136(9.9-1071)	112(6.9-891)	271(13.1-1802)	120(1.74-1075)	131(1.67-911)	43.8(2.05-253)	84.7(0.67-938)	619(15.5-3573)	Anatolaki and Tsitouridou 2009
The northern part of the inner Eastern Carpathians, Romania	Urban	47.05 (4.85-432.83)	34.90 (0.48-782.20)	173.32 (0.02-3667.50)	36 (1.26-1194.01)	54.45(1.11-359.95)	13.25(0.43-181.08)	28.10(2.88-239.46)	250.12(18.11-7440.49)	Keresztesi et al. 2020
	Urban	50.07(0.03-518.29)	33.71(1.85-188.49)	77.95(0.77-788.86)	35.95(2.16-409.75)	83.94(2.27-699.89)	24.56(1.59-268.30)	39.51(7.49-530.34)	148.95(2.25-1468.64)	

	Urban	57.80(2.43-331.31)	38.38(0.53-563.87)	84.13(0.50-815.72)	50.69(1.74-398.0)	101.68(5.99-3032.41)	39.49(2.05-503.09)	49.70(8.06-579.30)	185.22(3.19-1813.96)	
	Urban	58.78 (1.75-309.48)	36.72 (0.16-583.34)	75.98 (0.15-697.46)	38.38 (0.96-472.38)	81.23 (0.78-1905.65)	64.76 (2.92-1606.21)	40.28 (10.37-603.83)	148.75 (24.0-2012.58)	
US West coast	A coastal urban	24.96 (0.39-694.37)	2.85 (0.15-111.05)	6.50 (0.38-91.38)	19.53 (0.13-247.00)	3.34 (0.22-234.78)	0.64 (0.05-9.00)	2.81 (0.08-17.42)	3.16 (0.15-65.20)	
	A coastal urban	13.06 (0.25-80.56)	6.17 (0.74-125.47)	5.79 (0.29-83.06)	11.11 (0.04-70.91)	6.92 (0.33-214.83)	0.64 (0.05-9.00)	2.81 (0.08-17.42)	3.16 (0.15-65.20)	
	A coastal urban	28.12 (0.31-458.17)	30.89 (1.98-248.97)	15.10 (0.88-128.60)	25.82 (0.1-432.70)	27.01 (1.28-161.22)	1.18 (0.13-432.70)	6.87 (0.08-106.33)	10.36 (0.45-238.75)	
US East coast	A coastal urban near metropolitan cities	40.11 (1.18-361.75)	15.89 (1.00-115.71)	23.51 (1.46-202.56)	34.03 (0.57-314.87)	16.35 (0.44-108.00)	1.90 (0.05-53.44)	6.26 (0.33-71.50)	5.00 (3.76-6.47)	
	A coastal urban	33.30 (0.99-409.41)	11.82 (0.76-61.03)	19.01 (3.02-129.44)	28.34 (0.57-333.13)	7.17 (0.76-61.03)	0.99 (0.05-7.67)	6.85 (0.25-79.58)	5.62 (0.85-70.60)	
	A coastal urban	55.47 (5.21-918.55)	11.94 (5.21-918.85)	20.64 (1.44-125.10)	48.33 (4.30-809.39)	12.01 (0.11-57.24)	2.86 (0.13-46.03)	11.59 (0.58-180.00)	10.23 (0.90-148.80)	
the Hawaii	a coastal under high influence of local vulcanic emissions	7.47 (0.56-132.39)	2.43 (0.32-20.81)	25.86 (0.83-610.42)	13.96 (0.13-820.87)	3.23 (0.56-47.22)	0.75 (0.05-38.92)	3.39 (0.25-85.67)	5.10 (0.50-75.00)	Ma et al. 2021
the Bermuda	a coastal under high influence of anthropogenic emissions from North America	358.71 (8.35-19891.06)	8.34 (0.11-76.42)	47.35 (1.31-942.33)	305.11 (6.09-16670.29)	4.62 (0.11-50.34)	6.61 (0.08-332.48)	69.20 (1.73-3640.48)	17.36 (0.10-823.35)	
the Philippines	a coastal urban with biomass burning sources, vehicles, and industry	48.75 (3.78-2126.4)	33.99 (0.27-520.92)	71.71 (9.24-1088.90)	40.31 (3.35-1739.89)	253.26 (0.61-11006.10)	43.68 (0.26-1537.54)	23.19 (0.41-309.42)	57.66 (1.50-877.69)	

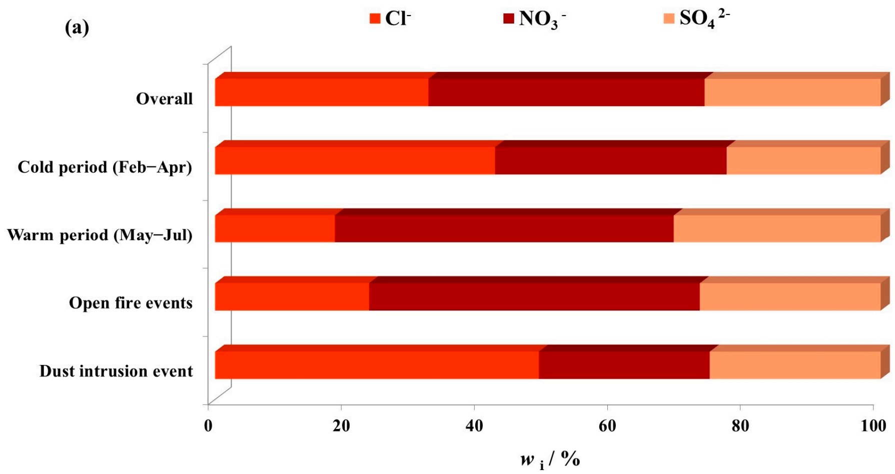

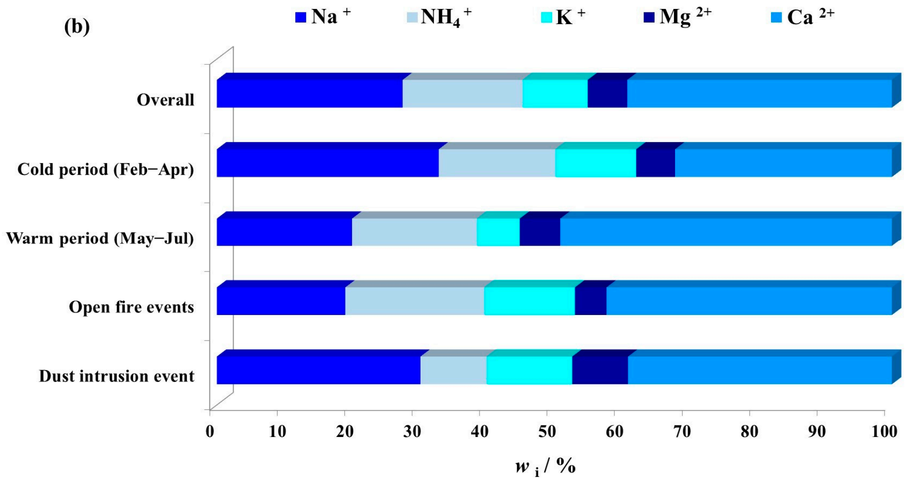

Figure S4. Average contributions of (a) each anion to the total anion flux and (b) each cation to the total cation flux determined in the bulk samples during the sampling campaign from February to July 2019 at the Middle Adriatic coastal site.

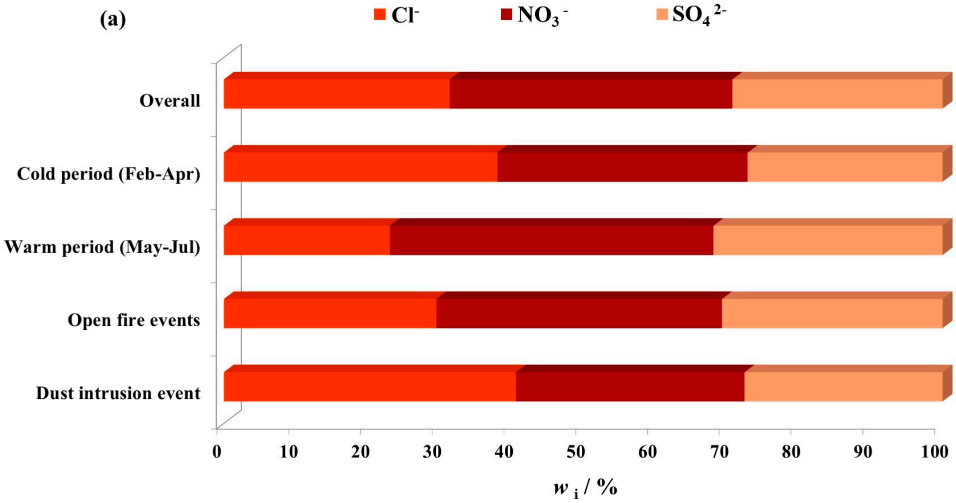

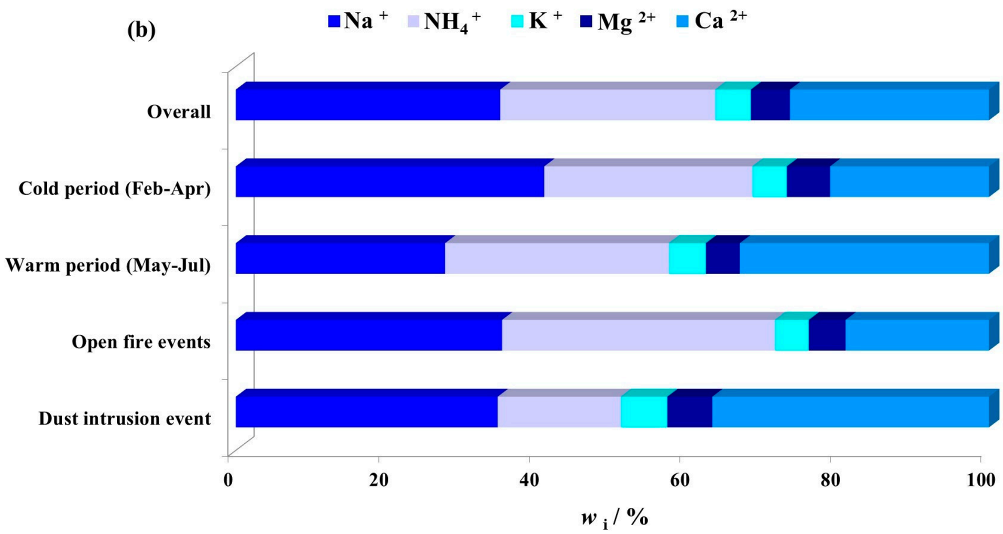

Figure S5. Average contributions of (a) each anion to the total anion flux and (b) each cation to the total cation flux determined in the wet deposition samples during the sampling campaign from February to July 2019 at the Middle Adriatic coastal site.

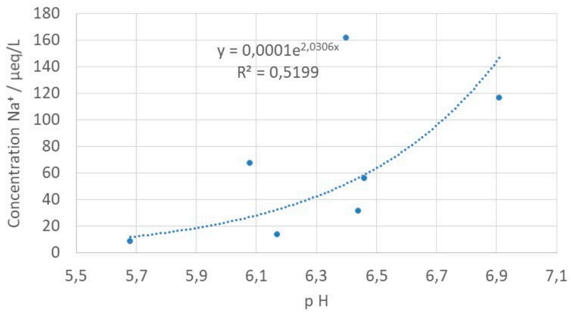

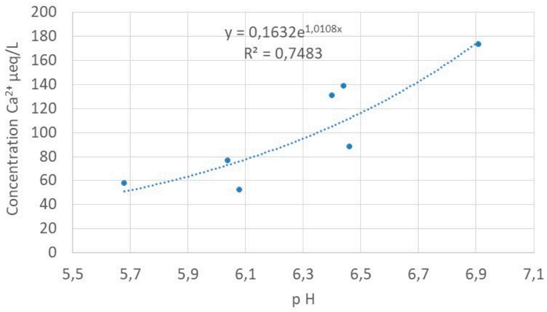

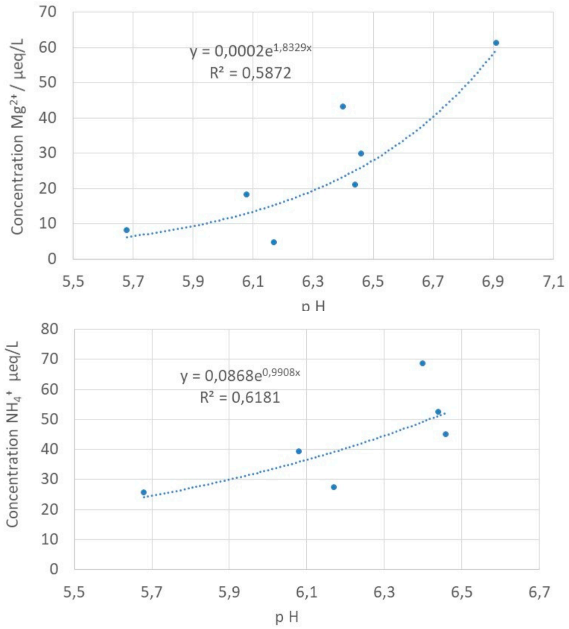

Figure S6. Relationship between pH and the equivalent concentrations of Na^+, Ca^{2+}, Mg^{2+} and NH_4^+ in bulk deposition samples.

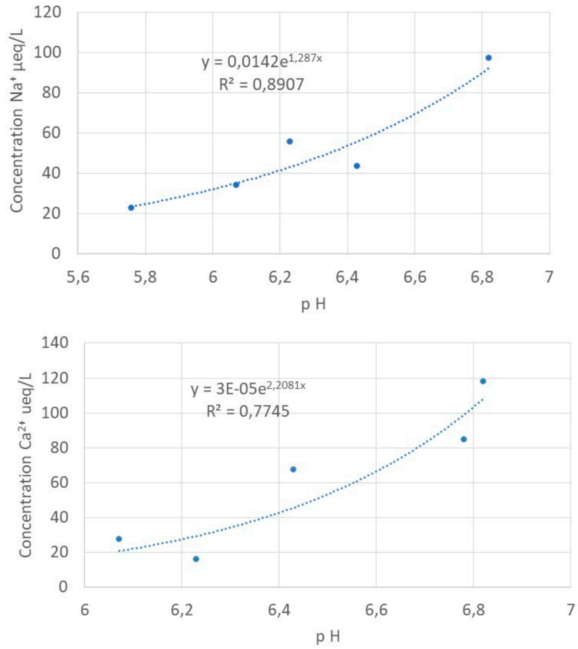

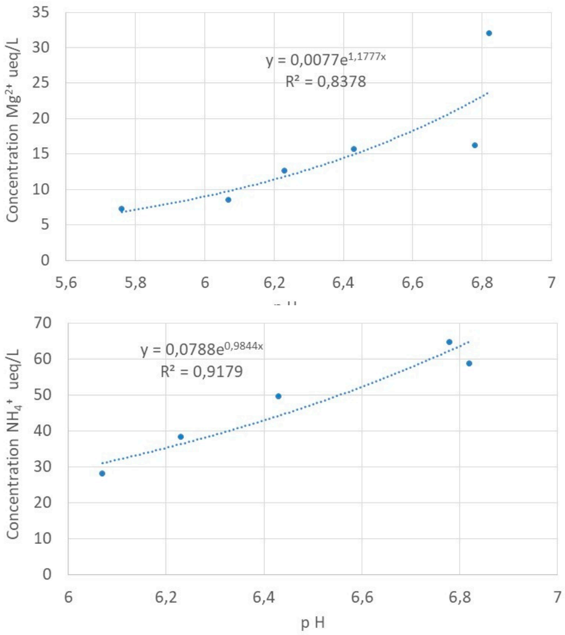

Figure S7. Relationship between pH and the equivalent concentrations of Na^+, Ca^{2+}, Mg^{2+} and NH_4^+ in wet deposition samples.

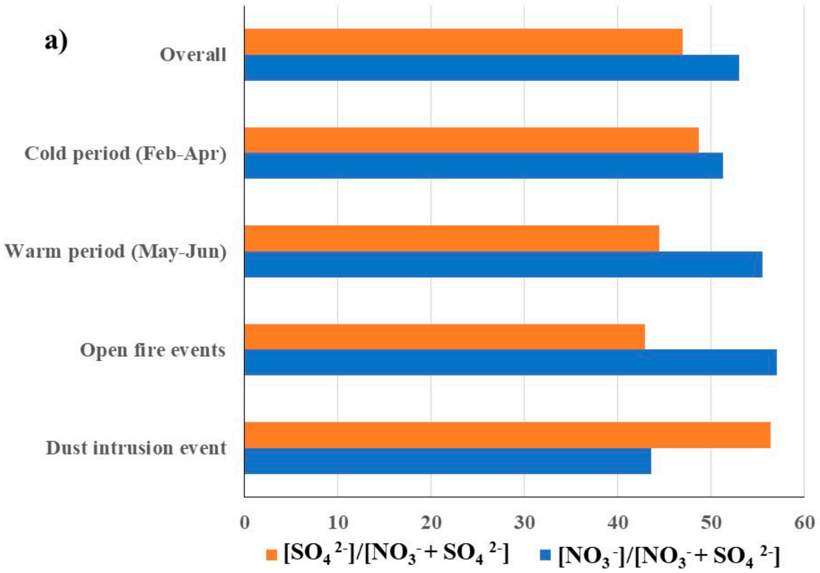

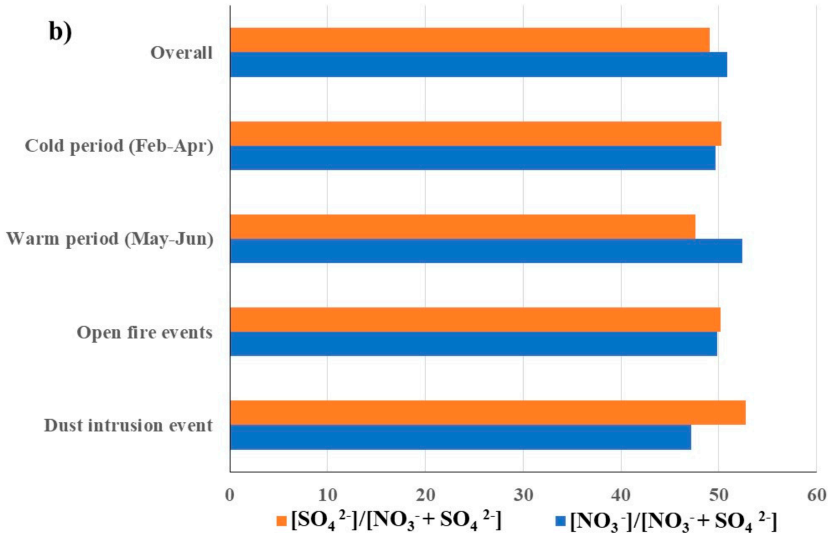

Figure S8. Average equivalent ratios; $[NO_3^-]$ / $[NO_3^- + SO_4^{2-}]$ and $[SO_4^{2-}]$ / $[NO_3^- + SO_4^{2-}]$ in (a) bulk deposition and (b) wet deposition for overall period, cold and warm seasons and special events.

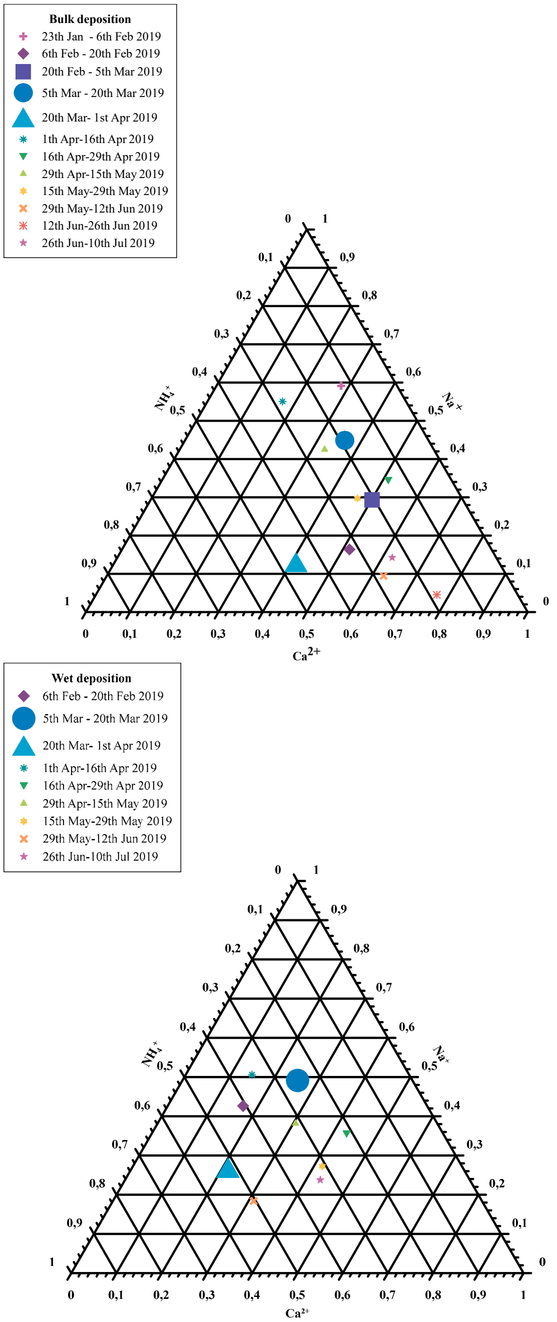

Figure S9. Relative proportion of major cation NFs in bulk and wet deposition.

Table S2. Comparison of multiple linear regression equations obtained in this study with similar studies.

Location, sample type	Linear regression equation	Reference
the Eastern Middle Adriatic coast Bulk deposition	$[NO_3^-] = 15.667 - 0.034[Na^+] + 0.544[NH_4^+] - 0.435[K^+] + 0.130[Mg^{2+}] - 0.001[Ca^{2+}]$<br>$[SO_4^{2-}] = 2.540 + 0.019[Na^+] + 0.548[NH_4^+] - 0.178[K^+] + 0.427[Mg^{2+}] - 0.042[Ca^{2+}]$<br>$[Cl^-] = 36.155 + 1.457[Na^+] - 1.202[NH_4^+] + 0.252[K^+] - 0.131[Mg^{2+}] - 0.147[Ca^{2+}]$	This study
the Eastern Middle Adriatic coast Wet deposition	$[NO_3^-] = 17.288 - 0.089[Na^+] + 0.304[NH_4^+] - 0.163[K^+] + 0.489 [Mg^{2+}] - 0.014[Ca^{2+}]$<br>$[SO_4^{2-}] = 23.397 - 0.779[Na^+] + 0.157[NH_4^+] + 2.534[K^+] + 3.475[Mg^{2+}] - 0.417[Ca^{2+}]$<br>$[Cl^-] = 2.188 - 0.156[Na^+] + 0.091[NH_4^+] + 0.365[K^+] - 5.075[Mg^{2+}] - 0.621[Ca^{2+}]$	This study
Central Anatolia,	$[NO_3^-] = -0.35 + 0.16[H^+] + 0.56[Ca^{2+}] + 0.24[NH_4^+] + 0.13[Na^+]$	[78]

rainwater	$[SO_4^{2-}] = 1.9 + 0.08[H^+] + 0.19[Ca^{2+}] + 0.11[NH_4^+] + 0.22[Mg^{2+}] + 0.15[Na^+]$	
Mediterranean, rainwater	$[NO_3^-] = -1.1 + 0.85[H^+] + 0.6[Ca^{2+}] + 0.72[NH_4^+]$<br>$[SO_4^{2-}] = 14 + 0.01[H^+] + 0.19[Ca^{2+}] + 0.14[NH_4^+] + 0.64[Mg^{2+}]$	[78]
Black Sea, rainwater	$[NO_3^-] = -7.1 + 0.22[H^+] + 0.11[Ca^{2+}] + 0.27[NH_4^+]$<br>$[SO_4^{2-}] = 1.4 + 0.06[H^+] + 0.15[Ca^{2+}] + 0.25[NH_4^+] + 0.04[Mg^{2+}]$	[78]
Shangai, China, rainwater	$[NO_3^-] = 0.311[NH_4^+] + 0.181[Ca^{2+}] + 15.602$<br>$[SO_4^{2-}] = 0.491[NH_4^+] + 0.342[Ca^{2+}] + 26.344$	[67]
Jabiru, Australia, rainwater	$[NO_3^-] = 1.71 + 0.14[H^+] + 0.18[NH_4^+] – 0.01[Na^+] – 0.77[K^+]$<br>$[SO_4^{2-}] = -3.93 + 0.30[H^+] + 0.01[NH_4^+] + 0.06[Na^+] + 0.74[K^+]$<br>$[Cl^-] = 2.71 + 0.13[H^+] + 0.66[NH_4^+] + 0.63[Na^+] + 0.54[K^+]$	[79]

Table S3. Correlation coefficients between ionic species in bulk and wet deposition at Martinska site; statistically significant correlations (P< 0.05) were marked red.

		**NO_3^-**	**SO_4^{2-}**	**Na^+**	**NH_4^+**	**K^+**	**Mg^{2+}**	**Ca^{2+}**
	Cl^-	0.603	0.850	0.966	0.669	0.553	0.876	0.273
	NO_3^-		0.824	0.711	0.809	0.448	0.620	0.460
	SO_4^{2-}			0.927	0.925	0.775	0.864	0.391
Bulk deposition	Na^+				0.804	0.662	0.916	0.351
	NH_4^+					0.879	0.693	0.254
	K^+						0.608	0.097
	Mg^{2+}							0.618
	Cl^-	0.645	0.931	0.999	0.929	0.846	0.858	0.116
	NO_3^-		0.715	0.645	0.747	0.395	0.426	0.194
	SO_4^{2-}			0.921	0.996	0.922	0.785	0.086
Wet deposition	Na^+				0.919	0.831	0.866	0.129
	NH_4^+					0.900	0.740	0.028
	K^+						0.766	-0.010
	Mg^{2+}							0.496

Table S4. Enrichment factors in bulk deposition samples.

	MARINE					CRUSTAL				
Measuring period	EF_m (Cl^-)	EF_m (K^+)	EF_m (Ca^{2+})	EF_m (Mg^{2+})	EF_m (SO_4^{2-})	EF_c (Cl^-)	EF_c (K^+)	EF_c (Na^+)	EF_c (Mg^{2+})	EF_c (SO_4^{2-})
23th Jan - 6th Feb 2019	1.24	1.82	10.84	1.34	2.27	974.26	0.17	3.68	1.14	30.68
6th Feb - 20th Feb 2019	0.96	4.28	71.49	1.44	9.38	113.84	0.06	0.56	0.19	19.18
20th Feb - 5th Mar 2019	1.18	2.50	38.80	1.39	4.19	259.15	0.06	1.03	0.33	15.80
5th Mar - 20th Mar 2019	0.81	2.08	18.32	1.17	2.49	378.44	0.11	2.18	0.59	19.85
20th Mar - 1st Apr 2019	1.04	94.62	71.56	2.88	12.03	123.40	1.31	0.56	0.37	24.60
1th Apr - 16th Apr 2019	0.86	15.13	7.01	1.14	2.93	1039.73	2.14	5.70	1.49	61.17
16th Apr - 29th Apr 2019	0.79	11.22	33.88	2.31	2.95	198.16	0.33	1.18	0.63	12.75
29th Apr - 15th May 2019	0.72	2.47	17.55	1.19	3.66	351.32	0.14	2.28	0.62	30.54
15th May -29th May 2019	0.68	4.49	35.61	2.33	5.19	162.57	0.13	1.12	0.60	21.33
29th May - 12th Jun 2019	0.56	25.20	150.92	4.09	21.03	31.53	0.17	0.26	0.25	20.38
12th Jun - 26th Jun 2019	0.42	45.94	389.42	11.87	26.98	9.11	0.12	0.10	0.28	10.14
26th Jun - 10th Jul 2019	0.71	11.06	99.36	2.91	10.53	60.94	0.11	0.40	0.27	15.50

Table S5. Enrichment factors in wet deposition samples.

	MARINE					CRUSTAL				
Measuring period	EF_m (Cl^-)	EF_m (K^+)	EF_m (Ca^{2+})	EF_m (Mg^{2+})	EF_m (SO_4^{2-})	EF_c (Cl^-)	EF_c (K^+)	EF_c (Na^+)	EF_c (Mg^{2+})	EF_c (SO_4^{2-})
6th Feb - 20th Feb 2019	1.02	2.26	8.88	1.25	4.42	975.78	0.25	4.50	1.29	72.75
5th Mar - 20th Mar 2019	0.79	2.04	11.78	1.13	2.54	573.75	0.17	3.39	0.88	31.50
20th Mar - 1st Apr 2019	0.77	3.60	17.95	1.18	5.84	363.03	0.20	2.23	0.60	47.64
1th Apr - 16th Apr 2019	0.75	2.85	6.58	1.00	5.23	964.99	0.43	6.07	1.40	116.31
16th Apr - 29th Apr 2019	0.74	4.72	27.61	1.45	3.54	226.74	0.17	1.45	0.48	18.75
29th Apr - 15th May 2019	0.73	3.16	18.15	1.10	4.79	340.10	0.17	2.20	0.56	38.63
15th May -29th May 2019	0.71	3.30	34.91	1.58	6.33	172.95	0.09	1.14	0.42	26.53
29th May - 12th Jun 2019	0.80	6.78	38.49	1.38	12.30	177.69	0.17	1.04	0.33	46.76
26th Jun - 10th Jul 2019	0.67	7.04	41.29	1.54	7.14	138.68	0.17	0.97	0.34	25.30

Section S1

Acidity and neutralization capacity of atmospheric deposition

Several approaches for assessing the acidification and neutralization capacity of bulk and wet deposition were applied and discussed in this study. As acidity of atmospheric deposition primary depends on the amount of most abundant anions, NO_3^- and SO_4^{2-}, the equivalent ratio $[H^+] / [NO_3^- + SO_4^{2-}]$ shows whether the acidity contributed by these strong anions is neutralized or not (Balasubramanian et al. 2001; Anatolaki and Tsitouridou 2009; Chen et al. 2020). This ratio is called fractional acidity (FA) and it would be equal to 1 if the acidity of atmospheric deposition caused by strong acids (H_2SO_4 and HNO_3) is not completely neutralized by alkaline cations, while deviations from 1 show the percentage level of neutralized acidic compounds. Furthermore, ratios of anion equivalent concentrations $[NO_3^-] / [NO_3^- + SO_4^{2-}]$ or $[SO_4^{2-}] / [NO_3^- + SO_4^{2-}]$ can be used to assess their contribution in the deposition acidification (Shen et al. 2012). On the other hand, the buffering ability of cations in atmospheric deposition can be estimated using the neutralization factor (NF_i) according to equation (1) (Anatolaki and Tsitouridou 2009; Calvo et al. 2010; Conradie et al. 2016; Ma et al. 2021; Pereira et al. 2021):

$NF_i = [X_i] / [nssSO_4^{2-} + NO_3^-]$ (S1)

where X_i is the equivalent concentration of alkaline species $[Na^+, Ca^{2+}, K^+, Mg^{2+}, NH_4^+]$ in μeq L^{-1} and $[SO_4^{2-} + NO_3^-]$ is the sum of equivalent concentrations of SO_4^{2-} and NO_3^-. Although this model may overestimate neutralization factors for alkaline species due to undetermined anions in the denominator such as bicarbonate or organic acids, it is well accepted in the literature to quantify its relative importance and magnitude of atmospheric deposition neutralization.

Many studies express acid-base relations between determined ionic compounds in atmospheric deposition through the ion balance (Shen et al. 2012; Xing et al. 2017; Meng et al. 2019; Ma et al. 2021). The ratio $\Sigma A^- / \Sigma K^+$ (sum of anion equivalents/ sum of cation equivalents) lower than 1 indicates the acidic properties of the deposition; however, greater deviation could indicate possible missing of parameters like HCO_3^- or organic acidic ions (Ma et al. 2021). Some authors (Anatolaki and Tsitouridou 2009; Keresztesi et al. 2019) therefore, suggest to calculate $[HCO_3^-]$ equivalent concentrations (in eq L^{-1}) by using equation (2):

$[HCO_3^-] = 10^{-11.24+pH}$ (S2)

Another method for estimation of the neutralizing capacity of alkaline ions over acidic ones is by calculating the ratio of neutralization potential (NP) to acidification potential (AP) according to the equation (3) (Safai et al. 2004; Chen et al. 2020). NP/AP ratio higher than 1 may indicate that the overall dominance of alkaline ionic species prevents the acidity of bulk deposition.

$NP/AP = [nssCa^{2+}] + [NH_4^+]/[nssSO_4^{2-}] + [NO_3^-]$ (S3)

The non-seasalt fractions $[nssCa^{2+}]$ and $[nssSO_4^{2-}]$ were calculated using methodology described in Keene et al. (1986) where the $nss[SO_4^{2-}]= [SO_4^{2-}]_{total} - ss[SO_4^{2-}]$ and $nss[Ca^{2+}]= [Ca^{2+}]_{total} - ss[Ca^{2+}]$ were calculated by subtracting the sea-salt fraction of each ion from the total measured amount. The sea-salt fraction was calculated from seawater ratio $ss[SO_4^{2-}]/[Na^+]=0.12$ and $ss[Ca^{2+}]/[Na^+]=0.04$. All concentrations are given in μeq L^{-1}.

Anatolaki and Tsitouridou (2009) and Meng et al., (2019) investigated the relationship between anions and cations using multiple linear regression analysis (MLR). The main acidic anions, SO_4^{2-} and NO_3^-, were considered as dependent variables, while cations Ca^{2+}, NH_4^+ and H^+, for which the highest linear correlation coefficients were obtained, were considered as independent variables.

All above mentioned models were applied and discussed in this study.

References

Anatolaki C, Tsitouridou R (2009) Relationship between acidity and ionic composition of wet precipitation. A two years study at an urban site, Thessaloniki, Greece. Atmos Res 92:100–113. https://doi.org/10.1016/j.atmosres.2008.09.008

Balasubramanian, R. Victor, T. Chun N (2001) Chemical and statistical analysis of precipitation in Singapore. Water Air Soil Pollut 130:451–456. https://doi.org/10.1023/A:1013801805621

Calvo AI, Olmo FJ, Lyamani H, et al (2010) Chemical composition of wet precipitation at the background EMEP station in Víznar (Granada, Spain) (2002-2006). Atmos Res 96:408–420. https://doi.org/10.1016/j.atmosres.2010.01.013

Cerro, J.C.; Cerdà, V.; Caballero, S.; Bujosa, C.; Alastuey, A.; Querol, X.; Pey, J. Chemistry of Dry and Wet Atmospheric Deposition over the Balearic Islands, NW Mediterranean: Source Apportionment and African Dust Areas. Sci. Total Environ. 2020, 747, 141187, doi:10.1016/j.scitotenv.2020.141187

Chen HY, Hsu LF, Huang SZ, Zheng L (2020) Assessment of the components and sources of acid deposition in northeast asia: A case study of the coastal and metropolitan cities in Northern Taiwan. Atmosphere (Basel) 11:. https://doi.org/10.3390/atmos11090983

Conradie EH, Van Zyl PG, Pienaar JJ, et al (2016) The chemical composition and fluxes of atmospheric wet deposition at four sites in South Africa. Atmos Environ 146:113–131. https://doi.org/10.1016/j.atmosenv.2016.07.033

Izquierdo, R.; Avila, A. Comparison of Collection Methods to Determine Atmospheric Deposition in a Rural Mediterranean Site (NE Spain). J. Atmos. Chem. 2012, 69, 351–368, doi:10.1007/s10874-012-9246-1

Kara, M.; Dumanoglu, Y.; Altiok, H.; Elbir, T.; Odabasi, M.; Bayram, A. Seasonal and Spatial Variations of Atmospheric Trace Elemental Deposition in the Aliaga Industrial Region, Turkey. Atmos. Res. 2014, 149, 204–216, doi:10.1016/j.atmosres.2014.06.009

Keene WC, Pszenny AAP, Galloway JN, Hawley ME (1986) Sea-salt corrections and interpretation of constituent ratios in marine precipitation. J Geophys Res 91:6647. https://doi.org/10.1029/jd091id06p06647

Keresztesi Á, Birsan MV, Nita IA, et al (2019) Assessing the neutralisation, wet deposition and source contributions of the precipitation chemistry over Europe during 2000–2017. Environ Sci Eur 31:1–15. https://doi.org/10.1186/s12302-019-0234-9

Keresztesi, Á.; Nita, I.A.; Birsan, M.V.; Bodor, Z.; Pernyeszi, T.; Micheu, M.M.; Szép, R. Assessing the Variations in the Chemical Composition of Rainwater and Air Masses Using the Zonal and Meridional Index. Atmos. Res. 2020, 237, 104846, doi:10.1016/j.atmosres.2020.104846

Ma L, Dadashazar H, Hilario MRA, et al (2021) Contrasting wet deposition composition between three diverse islands and coastal North American sites. Atmos Environ 244:117919. https://doi.org/10.1016/j.atmosenv.2020.117919

Meng Y, Zhao Y, Li R, et al (2019) Characterization of inorganic ions in rainwater in the megacity of Shanghai: Spatiotemporal variations and source apportionment. Atmos Res 222:12–24. https://doi.org/10.1016/j.atmosres.2019.01.023

Morera-Gómez, Y.; Santamaría, J.M.; Elustondo, D.; Lasheras, E.; Alonso-Hernández, C.M. Determination and Source Apportionment of Major and Trace Elements in Atmospheric Bulk Deposition in a Caribbean Rural Area. Atmos. Environ. 2019, 202, 93–104, doi:10.1016/j.atmosenv.2019.01.019

Pereira JN, Fornaro A, Vieira-Filho M (2021) Source Apportionment of Atmospheric Deposition Species in an Agricultural Brazilian Region Using Positive Matrix Factorization. 12. https://doi.org/10.3390/ecas2021-10698

Pieri, L.; Matzneller, P.; Gaspari, N.; Marotti, I.; Dinelli, G.; Rossi, P. Bulk Atmospheric Deposition in the Southern Po Valley (Northern Italy). Water. Air. Soil Pollut. 2010, 210, 155–169, doi:10.1007/s11270-009-0238-y

Rossini, P.; Guerzoni, S.; Molinaroli, E.; Rampazzo, G.; De Lazzari, A.; Zancanaro, A. Atmospheric Bulk Deposition to the Lagoon of Venice: Part I. Fluxes of Metals, Nutrients and Organic Contaminants. Environ. Int. 2005, 31, 959–974, doi:10.1016/j.envint.2005.05.006

Safai PD, Rao PSP, Momin GA, et al (2004) Chemical composition of precipitation during 1984-2002 at Pune, India. Atmos Environ 38:1705–1714. https://doi.org/10.1016/j.atmosenv.2003.12.016

Shen Z, Zhang L, Cao J, et al (2012) Chemical composition, sources, and deposition fluxes of water-soluble inorganic ions obtained from precipitation chemistry measurements collected at an urban site in northwest China. J Environ Monit 14:3000–3008. https://doi.org/10.1039/c2em30457k

Xing J, Song J, Yuan H, et al (2017) Chemical characteristics, deposition fluxes and source apportionment of precipitation components in the Jiaozhou Bay, North China. Atmos Res 190:10–20. https://doi.org/10.1016/j.atmosres.2017.02.001
